# A Ternary Flavonoid Formulation Mitigates Fractional Radiation-Induced Brain Injury via Transcriptomic Reprogramming and Synaptic Protection

**DOI:** 10.3390/ijms27177721

**Published:** 2026-08-28

**Authors:** Yuanbing Zhu, Yishu Yin, Ting Ju, Heqi Gao, Jiayu Wang, Fangjing Miao, Weihong Lu

**Affiliations:** 1 School of Chemistry and Chemical Engineering, Harbin Institute of Technology, Harbin 150001, China; zhuyb27@163.com (Y.Z.); ab873460057@163.com (T.J.); wangjiayu9819@163.com (J.W.); 2 National and Local Joint Engineering Laboratory for Synthesis, Harbin Institute of Technology, Harbin 150001, China; yishuyin1113@163.com (Y.Y.); ghq10200@126.com (H.G.); mmfj1002@163.com (F.M.); 3 Zhengzhou Advanced Research Institute, Harbin Institute of Technology, Zhengzhou 450000, China

**Keywords:** fractional radiation, transcriptomics, synaptic plasticity, flavonoids, neuroprotection

## Abstract

Fractional ionizing radiation (FIR) is a standard cancer therapy but often induces severe central nervous system complications, including cognitive decline and physiological dysfunction. While natural flavonoids hold therapeutic promise for radiation-induced brain injury (RIBI), optimizing their combinations and elucidating the underlying molecular pathways remain challenging. Methods: To develop a precise therapeutic strategy, we first integrated network pharmacology and UHPLC-Q-Orbitrap MS/MS analysis to identify three highly effective flavonoid monomers from a radioprotective botanical extract. Subsequently, an in vivo anti-inflammatory screening was conducted to determine the optimal combinatorial ratio, designated as the ternary formulation QLI. The neuroprotective efficacy of QLI was then systematically evaluated in a mouse model of fractional RIBI (cumulative dose of 12 Gy) through behavioral assessments, hematopoietic profiling, and neurotransmitter analyses. Transcriptomic alterations were explored via RNA-sequencing (RNA-seq) and validated by molecular docking, RT-qPCR, and Western blotting. Results: Pharmacological and mass spectrometry analyses identified Quercetin, Luteolin, and Isorhamnetin-3-O-glucoside as the core bioactive monomers. Quantitative synergistic screening established the optimal QLI formulation at a mass ratio of 2:1:1. In vivo, FIR exposure induced severe spatial memory deficits, disrupted neurotransmitter homeostasis, and caused hematopoietic decline. Administration of QLI successfully reversed these physiological and cognitive impairments. Transcriptomic profiling revealed that QLI globally reprogrammed aberrant gene expression, specifically normalizing signaling networks related to the PI3K–Akt pathway, apoptosis, and neuroactive ligand–receptor interactions. Multidimensional validation confirmed that QLI mitigated neuroinflammation, suppressed astrocyte hyperactivation (GFAP), and preserved synaptic plasticity by preventing the pathological accumulation of SynGAP and autophagic stress (Beclin-1). Conclusions: The rationally designed ternary flavonoid formulation (QLI) provides potent neuroprotection against fractional RIBI. By resolving neuroinflammation, alleviating synaptic plasticity suppression, and normalizing stress-induced transcriptomic disruptions, QLI represents a promising multi-target experimental formulation with the potential to mitigate radiotherapy-associated neurological side effects.

## 1. Introduction

Fractionated radiotherapy (FRT) is a cornerstone strategy for treating primary and metastatic brain tumors in humans. By increasing the number of fractionation sessions (e.g., from 4–6 to 30 sessions), FRT effectively improves tumor curability while attempting to exploit the protective effect of fractionation on normal tissue [1]. However, healthy brain tissue is inevitably exposed to radiation during this process. Consequently, many patients experience severe side effects, such as headache, nausea, and progressive learning and memory deficits, which profoundly affect their quality of life and treatment compliance [2,3]. These symptoms are closely related to lesions in the central nervous system. Radiobiological experiments have shown that radiation-induced brain injury (RIBI) is dependent on various factors, including primary and secondary injury, early blood–brain barrier disruption, edema formation, white matter necrosis, vascular anomalies, and glial cell demyelination [4]. Specifically, radiation particles penetrating the brain interact with astrocytes, microglia, and neurons, triggering intense neuroinflammatory cascades and oxidative stress [5]. Appropriate animal models are essential for investigating the mechanisms of RIBI and evaluating potential therapeutics. Despite the life-prolonging benefits of conventional radiotherapy, the mitigation of RIBI remains a significant clinical challenge [6]. In the field of RIBI, the development of less toxic, multi-target nutraceuticals from food to be used concurrently with FRT, which are being rigorously evaluated and are expected to be used in combination therapies at all stages of RIBI, is improving [7]. Large single doses of radiation are not commonly used in clinical practice. FRT is the primary mode of radiation delivery in humans. The effects of various doses, fractional regimens, and irradiation times on histopathological and functional changes have been previously reported in rodents. Brown et al. [8] adopted a persistent low-dose fractional regimen in which rats were administered 5 Gy gamma rays twice a week and a total dose of 40 Gy to the whole brain for 4 weeks. The doses received during a trip to Mars will be high, up to 2 Gy for a 3-year mission, and will consist of highly biologically active rays [9]. Furthermore, occupational or accidental radiation exposure scenarios often range from 1.5–3.5 Gy [10]. In clinical settings, a fractional dose of 2 Gy is the most widely adopted standard for conventional human radiotherapy regimens [11]. On the basis of previous studies by our group simulating space radiation [12], and aiming to emulate the cumulative biological effects of these standard clinical fractions in a controlled experimental setting, we established a cumulative fractionated radiation (FIR) model using 2 Gy × 6 sessions in rodents. This regimen was designed to accurately reflect the pathological progression of functional and histopathological changes induced by repeated radiation exposure, while avoiding the acute lethality associated with massive single doses.

Compared with synthetic single-target drugs, natural products have attracted increasing attention owing to their multi-component synergistic effects and relatively low toxicity [13]. In this context, Xinwei Kangfu (XW) has predicted promising radioprotective properties. Preliminary studies focusing on the structural–functional relationships of dietary flavonoids elucidated the robust free-radical scavenging capacities of flavonols (such as quercetin) and their specific binding dynamics with blood carrier proteins, which critically dictate their systemic transport and bioavailability [14,15], and XW can protect cerebral vessels, modulate neurotransmitter biosynthesis, and inhibit radiation-induced apoptosis [16,17,18]. However, the complex chemical composition of crude botanical extracts often hinders precise mechanistic elucidation and clinical standardization.

Building upon this strong foundation and our preliminary phytochemical screening which revealed a flavonoid-rich fraction in XW, we aimed to develop a precise therapeutic formulation. Based on our network pharmacology analysis, Quercetin (Q), Luteolin (L), and Isorhamnetin-3-O-glucoside (I) were selected as the core monomers (designated as QLI). Quercetin provides broad-spectrum systemic antioxidant protection by activating the Nrf2 signaling pathway and upregulating BMI-1 to promote epithelial regeneration [19]. Luteolin specifically targets the vascular microenvironment, mitigating radiation-induced vascular injury by binding to HSPB1 to inhibit endothelial cell ferroptosis, while also modulating p53-mediated apoptosis [20]. Concurrently, Isorhamnetin penetrates the nucleus to actively facilitate DNA damage repair by promoting ATM phosphorylation and 53BP1 recruitment, which has been shown to rescue lethal gastrointestinal syndromes [21]. Together, we hypothesize that the QLI formulation may establish a synergistic, three-dimensional defense network—spanning from the cell membrane to the nucleus—potentially offering a comprehensive and scientifically grounded strategy for clinical radioprotection.

Given the complex synergistic interactions and multi-target nature of such ternary combinations, conventional pharmacological methods are often insufficient to comprehensively capture their systemic effects. To bridge this gap, we employed an integrated multi-omics approach. In this study, we first utilized computational network pharmacology and molecular docking to predict the active ingredients of XW and their potential targets in RIBI [22]. We then conducted in vivo RNA-seq on the brain tissues of irradiated mice to map the global transcriptomic reprogramming induced by the formula. By combining in silico predictions with transcriptomic profiling and experimental validation, this study aimed to systematically elucidate the molecular mechanisms through which QLI potentially alleviates fractional radiation-induced brain injury and to test the hypothesis of its multi-target synergy.

## 2. Results

### 2.1. Network Pharmacology Predicts Core Targets and Pathways of XW Against RIBI

**Figure A1:**
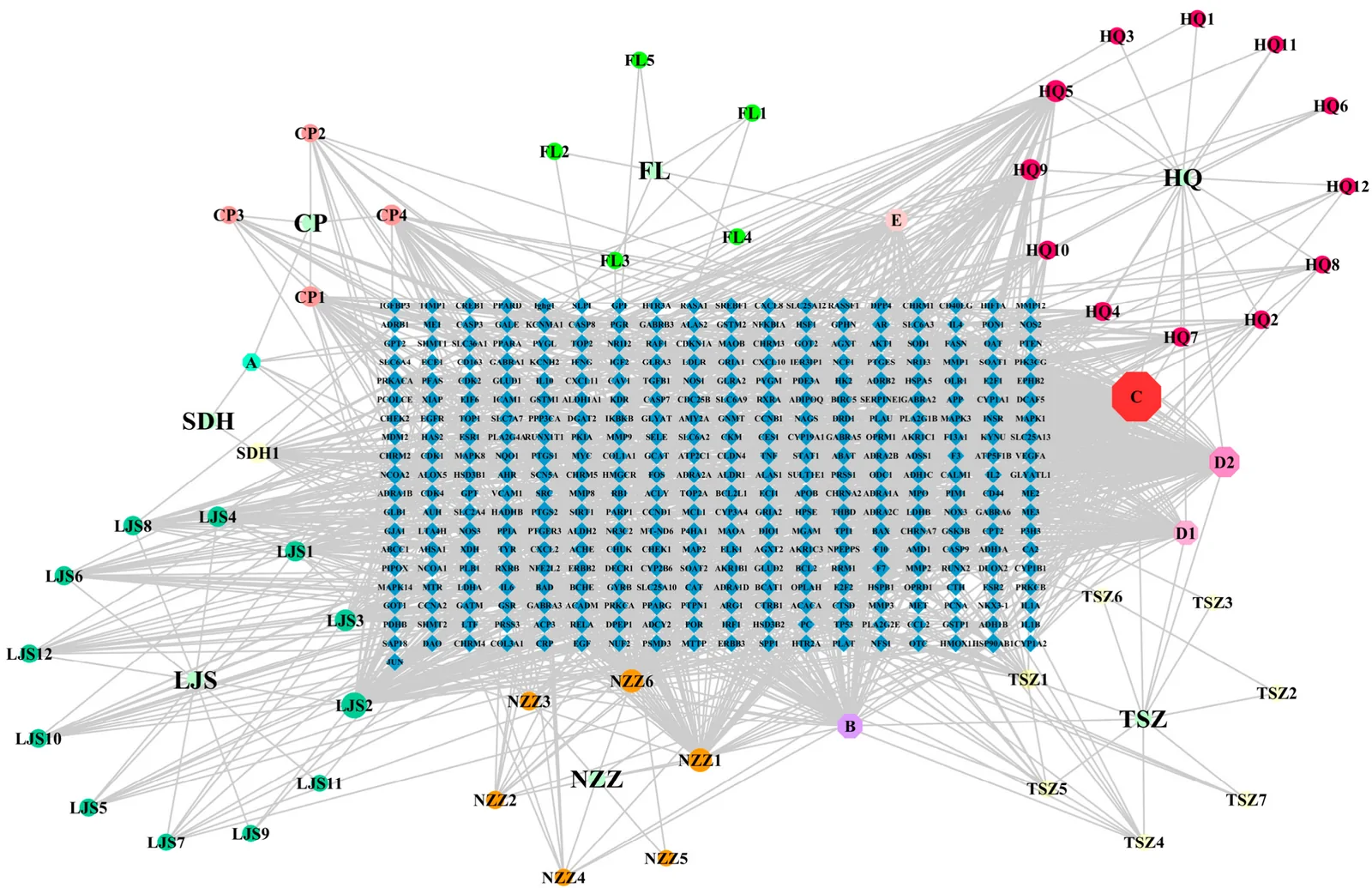
Effective Ingredient-Target Network.

The overlapping components are denoted by the same symbol; 52 components were chosen as candidate bioactive components for further analyses, and detailed information is shown in Table A2. The network and type files were then input into Cytoscape (v3.7.2) to construct a network of active ingredients and corresponding targets of XW, as shown in Figure A1.

**Figure 1:**
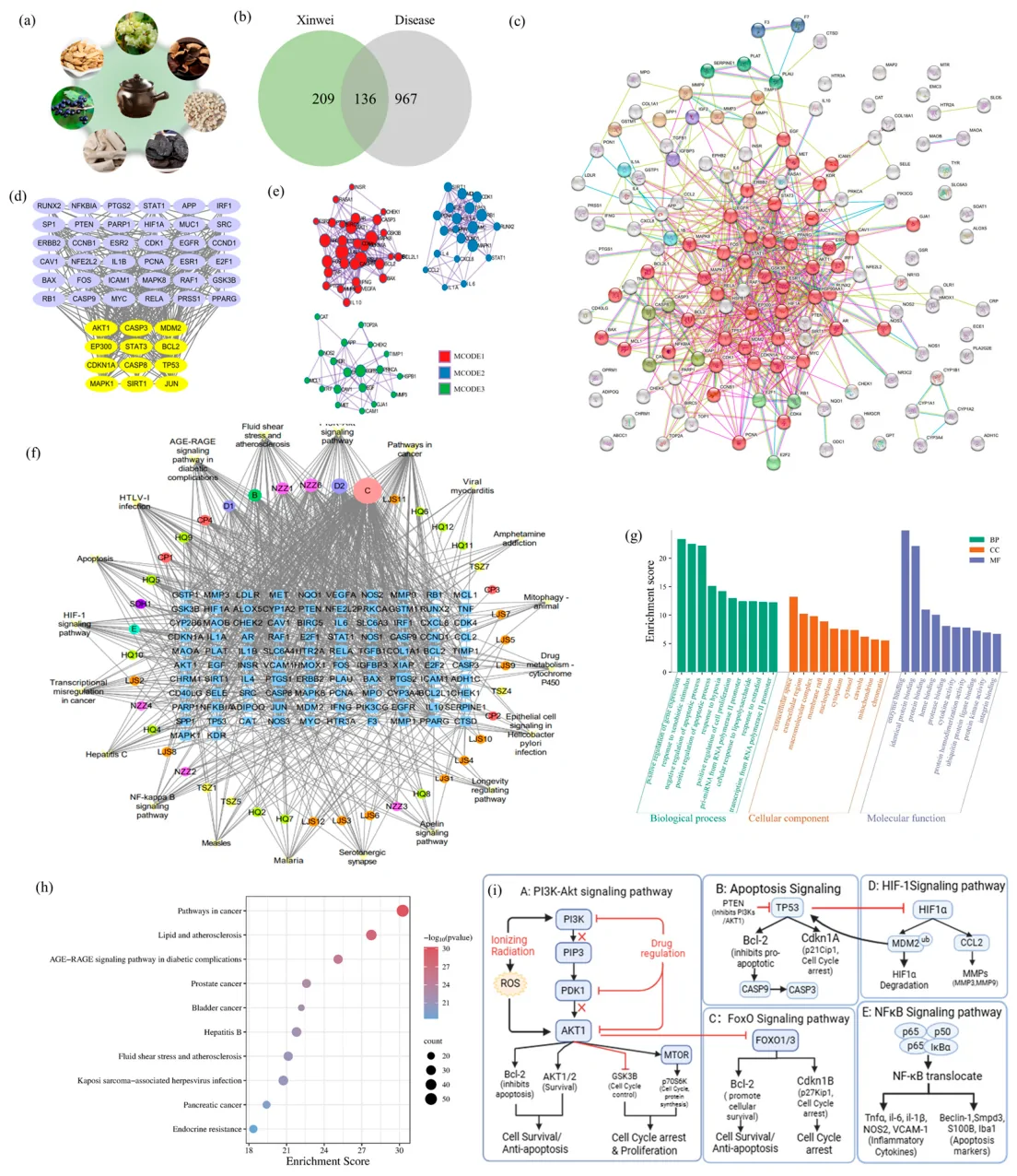
Network pharmacology analysis was used to predict the core targets and pathways through which XW acts against RIBI. (**a**) Representative images of the seven herbal constituents of XW; (**b**) Venn diagram showing the intersection of XW-predicted targets and RIBI-related targets; (**c**) protein-protein interaction (PPI) network of the 136 overlapping targets; (**d**) the top core hub genes extracted from the PPI network; (**e**) the top three cluster modules (MCODE1-3) identified in the PPI network. (**f**) Herb-Component-Target-Pathway multidimensional network; (**g**) Gene Ontology (GO) enrichment analysis of the common targets, including biological process (BP), cellular component (CC), and molecular function (MF) terms; (**h**) bubble plot showing the top KEGG signaling pathways; (**i**) schematic representation of the predicted primary mechanisms (e.g., the PI3K-AKT and AGE-RAGE signaling pathways) involved in the neuroprotective effect of XW.

In total, 7920 targets of radiation-induced brain damage were identified from the database. Duplicate values were deleted after the data were merged, and ultimately, 1103 radiation disease-related targets were obtained. Using a Venn diagram (Figure 1b), we matched the 345 genes predicted by XW and the 1103 genes predicted by RIBI disease to obtain 136 overlapping genes, which are likely the most critical genes for XW to act on RIBI disease. The targets were submitted to the STRING11.0 platform to construct a protein–protein interaction (PPI) network (Figure 1c).

The Metascape platform was used to analyze the signaling pathways associated with XW treatment of RIBI-related targets (Figure 1f). CytoHubba ranks nodes according to their network properties. This algorithm selects the MCC and displays the shortest path to it. The top 10 critical genes, AKT1, TNF, TP53, IL6, CASP3, IL1B, ESR1, JUN, BCL2, and PTGS2, played key roles (Table 1). Six genes were enriched in the AGE-RAGE and PI3K-AKT signaling pathways (Figure 1i).

We uploaded 136 genes common to XW and RIBI to DAVID for GO and KEGG analyses. The enrichment of BP, CC, and MF terms is displayed, and the top 10 enrichment scores are shown in Figure 1g. These pathways are closely related to many cancer-like processes; therefore, we believe that XW possibly acts on these pathways [23,24].

The core components and targets of XW for RIBI were analyzed using the built-in Network Analyzer of Cytoscape (v 3.7.2) (Figure 1f). Network analysis revealed that quercetin had a degree of 154 and a betweenness centrality of 0.32, suggesting that quercetin was the main ingredient in XW for the RIBI treatment (Table 2).

### 2.2. Qualitative Analysis of XW via UHPLC–LTQ–Orbitrap MS

**Figure 2:**
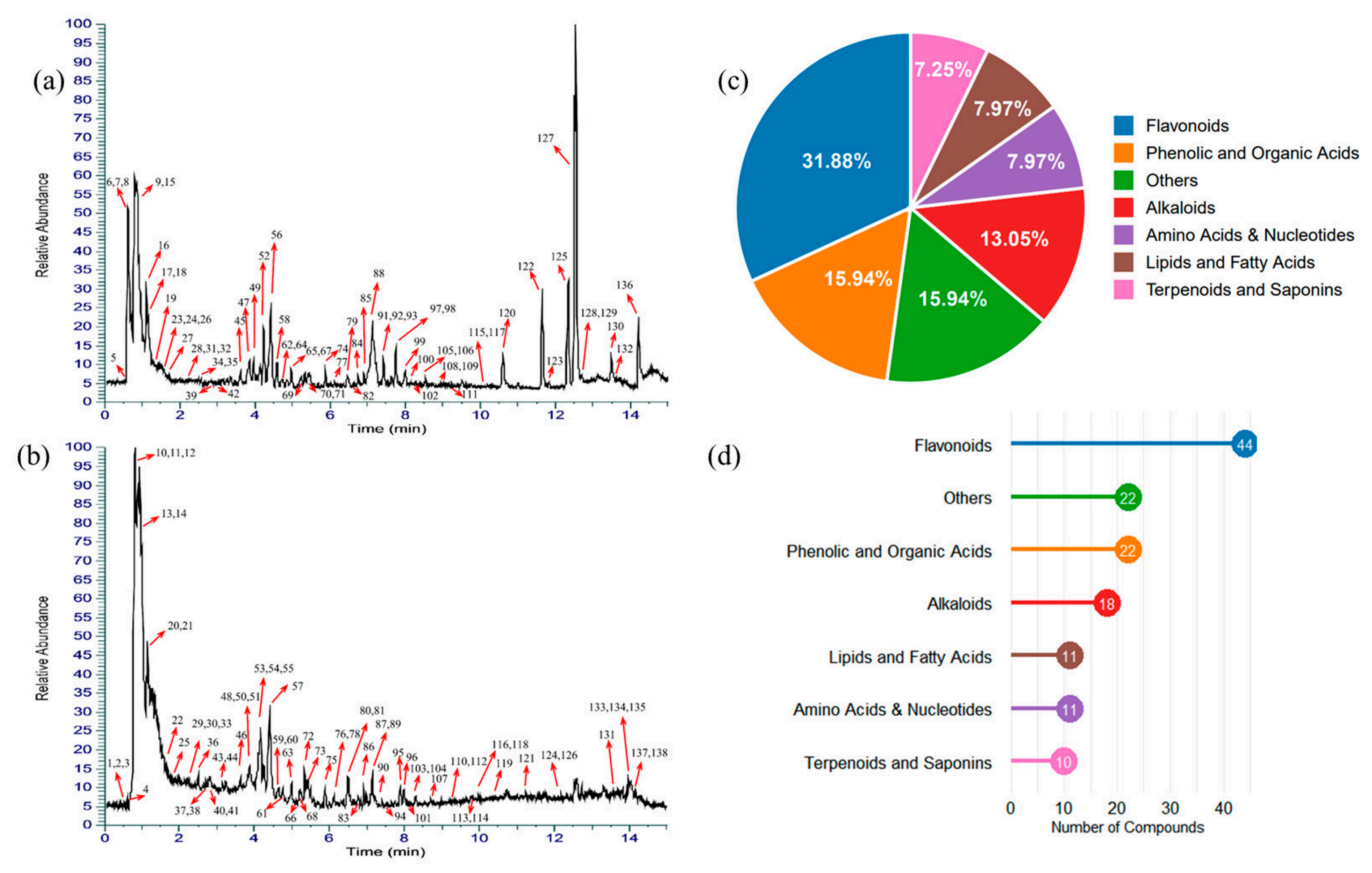
Comprehensive chemical characterization of XW extract by UHPLC-Q-Orbitrap MS/MS. Total ion chromatograms (TIC) in positive (**a**) and negative (**b**) ion modes. (**c**) Pie chart showing the percentage distribution of 138 identified compounds by major chemical classes. (**d**) Lollipop chart illustrating the precise number of compounds identified within each major category. The flavonoids and phenolic acids were found to be the predominant bioactive constituents.

**Figure 3:**
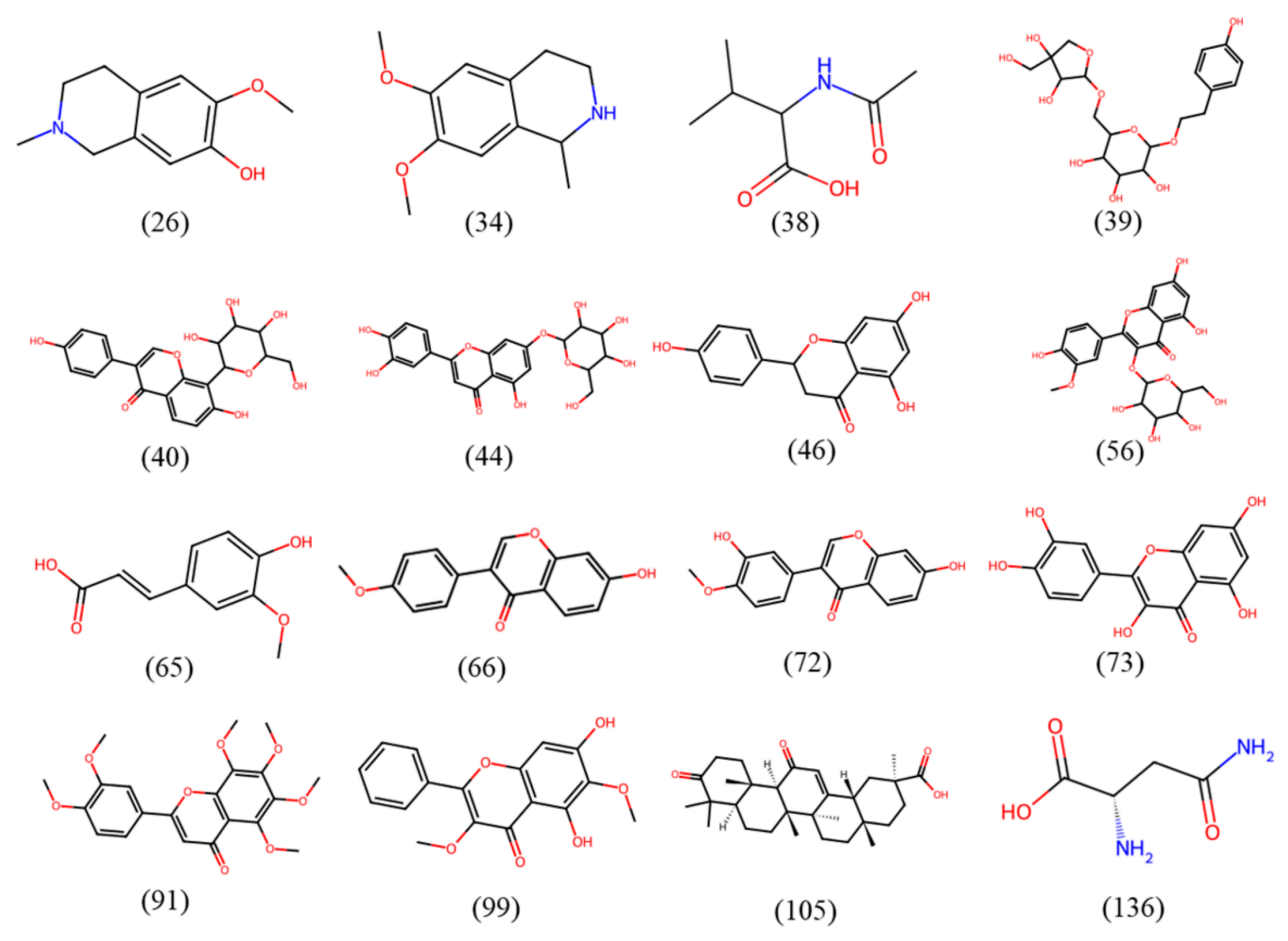
Structures of the representative components quantified in this study, selected on the basis of network pharmacology, analytical feasibility, and Chinese Pharmacopoeia requirements: (26) Corypalline, (34) (S)-Salsolidine, (38) N-Acetyl-DL-valine, (39) Osmanthuside H, (40) Daidzein-8-C-glucoside, (44) Luteolin-7-O-glucoside, (46) Naringenin, (56) Isorhamnetin-3-O-glucoside, (65) Ferulic Acid, (66) Formononetin, (72) Calycosin, (73) Quercetin, (91) Nobiletin, (99) 5,7-Dihydroxy-3,6-dimethoxyflavone, (105) 3-oxoglycyrrhetinic acid, and (136) L-Asparagine.

As summarized from the UHPLC–LTQ–Orbitrap/MS analysis, a total of 138 bioactive constituents were structurally identified in the XW extract (Table 3). These compounds can be broadly classified into several major categories, predominantly comprising flavonoids (44 compounds, including flavones, flavonols, and isoflavones), phenolic and organic acids (22 compounds), alkaloids (18 compounds), and terpenoids/saponins (10 compounds), as shown in Figure 2 and Figure 3. The high abundance of flavonoids and phenolic acids provides a material basis for the potent antioxidant and anti-neuroinflammatory efficacies observed in the RIBI model, which is highly consistent with our subsequent network pharmacology predictions (Figure 1).

**Figure 4:**
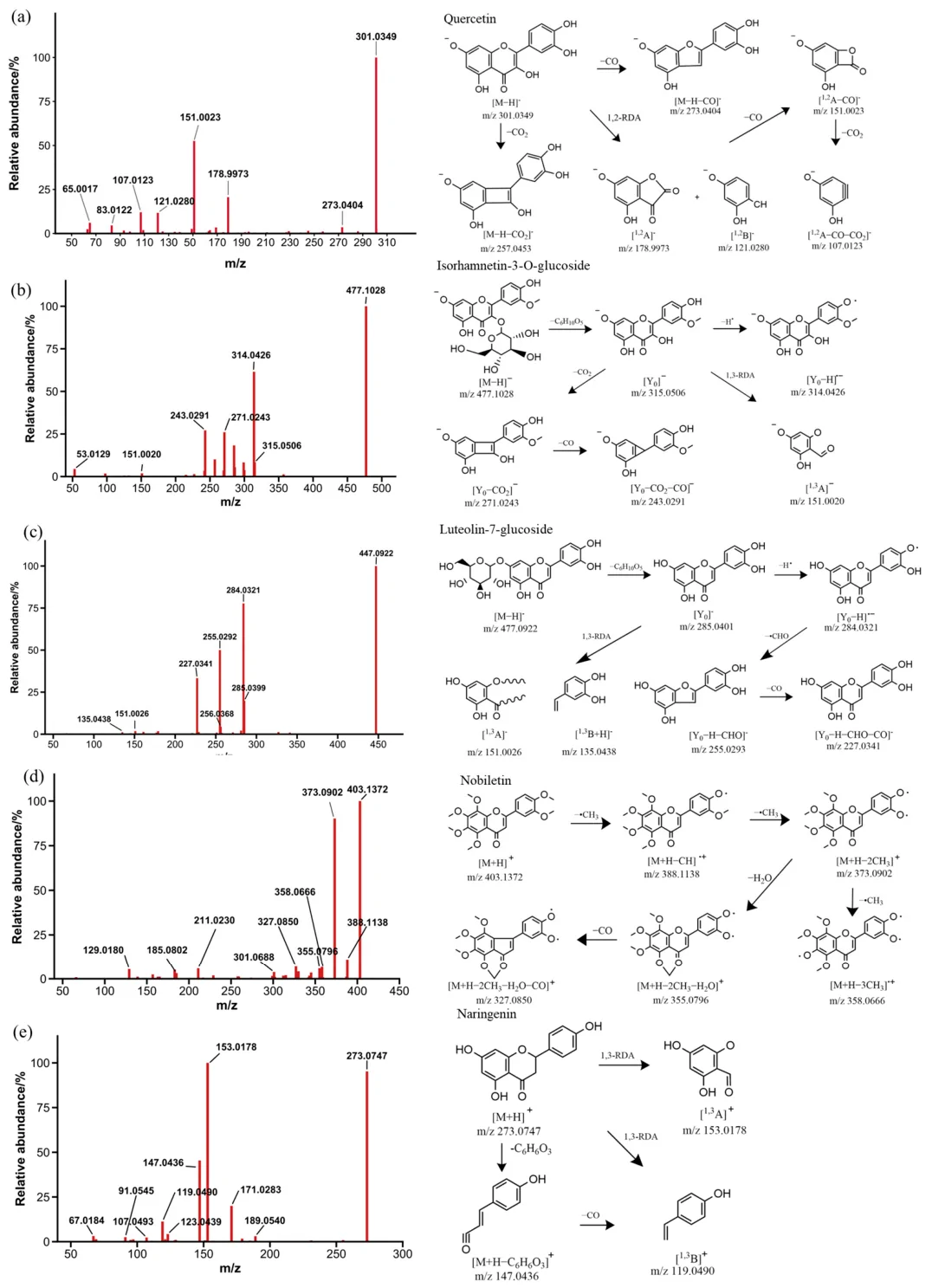
Proposed fragmentation pathway of (**a**) quercetin; (**b**) isorhamnetin-3-O-glucoside; (**c**) luteolin-7-glucoside; (**d**) nobiletin; and (**e**) naringenin.

Five flavonols were extracted mainly from XW, including Peaks 56, 73, 78, 84 and 99. In negative electrospray ionization (ESI-) mode, the precursor ion of Peak 73 (Figure 4a) was observed at *m*/*z* 301.0350, with [M-H-CO]^−^) and carbon dioxide ([M-H-CO2]^−^, -44Da) from the precursor ion. Under high-energy collision-induced dissociation (CID), the secondary fragment at *m*/*z* 107.0125 ([1,3A-CO2]^−^) was observed. Based on the precursor and product ion profiles, and in accordance with established mechanisms for dihydroflavonols and flavonols [16,17], peak 73 was identified as quercetin. Peak 56 exhibited a precursor ion at *m*/*z* 477.1035 ([M-H]^−^), suggesting a molecular formula of C22H22O12 (Figure 4b). Tandem mass spectrometry revealed a prominent aglycone ion at *m*/*z* 315.0512, resulting from the loss of a hexose moiety ([M-H-Glc]^−^, -162Da). Notably, a radical anion signal was observed at *m*/*z* 300.0276, originating from the homolytic cleavage of the 3′-methoxy group on the aglycone to release a methyl radical ([M-H-Glc-CH3·, -15Da). Further fragmentation of the skeleton yielded *m*/*z* 271.0233 ([M-H-Glc-CH3·-CHO]^−^) and the classical [1,3A]^−^ fragment at *m*/*z* 151.0025. Based on the loss of the hexose and the competitive radical cleavage of the methyl group [18], Peak 56 was identified as isorhamnetin-3-O-glucoside.

Flavones represent a major subclass of flavonoids characterized by a C6-C3-C6 diphenylpropane backbone, specifically featuring a double bond between the C-2 and C-3 positions and a carbonyl group at C-4 on the C-ring, of which 17 were present in XW, including Peaks 44, 55, 58, 62, 63, 64, 69, 74, 75, 89, 90–94, 100 and 109. Compound 44 displayed a deprotonated molecule [M-H]^−^ at *m*/*z* 447.0928 in ESI- mode, consistent with the molecular formula C21H20O11 (Figure 4c). In the MS/MS spectrum, the precursor ion underwent rapid heterolytic cleavage of the O-glycosidic bond, yielding a base peak at *m*/*z* 285.0389. This signal corresponds to the neutral loss of a glucose residue ([M-H-Glc]^−^), revealing the aglycone core. Subsequent fragmentation of the aglycone produced key ions at *m*/*z* 151.0025 and *m*/*z* 133.0288, identified as [1,3A]^−^ and [1,3B]^−^ RDA fragments, respectively. The absence of the [1,2A]^−^ fragment, in contrast to quercetin, confirmed the lack of a hydroxyl group at the C-3 position. Based on the neutral loss of the glycone and the specific RDA ion patterns [19], Compound 44 was characterized as luteolin-7-O-glucoside. Peak 91 showed optimal response in positive ionization (ESI+); following the extensive stripping of methoxy groups, the heterocyclic skeleton underwent RDA cleavage to produce [1,3A]+ at *m*/*z* 211.0237 and [1,3B]+ at *m*/*z* 163.0754. Correlating these radical neutral losses and specific product ions with literature data on biotransformation and pharmacokinetics [20], it was identified as nobiletin (Figure 4d).

Flavanones possess a distinct structure from flavones and flavonols, characterized by the absence of a double bond between C2 and C3. This subclass in XW includes Peaks 46, 52, 61, 62, 68 and 88. The precursor ion of Peak 46 appeared at *m*/*z* 271.0612 ([M-H]^−^) in ESI- mode, with the formula C15H12O5 (Figure 4e). The MS/MS spectrum was dominated by a pair of complementary and characteristic RDA fragments: [1,3A]^−^ at *m*/*z* 151.0031 and [1,3B]^−^ at *m*/*z* 119.0497. Secondary pathways included the direct loss of water ([M-H-H_2_O]^−^, *m*/*z* 253.0501) and carbon dioxide ([M-H-CO_2_]^−^, *m*/*z* 227.0701) from the precursor ion, as well as the further loss of CO2 from the [1,3A]^−^ fragment to form the ion at *m*/*z* 107.0125. By integrating these specific RDA patterns and neutral loss characteristics with high-resolution Orbitrap metadata [25], it was confirmed as naringenin.

### 2.3. Optimization of the Ternary Formulation and Evaluation of Its Synergistic Anti-Inflammatory Efficacy

**Figure 5:**
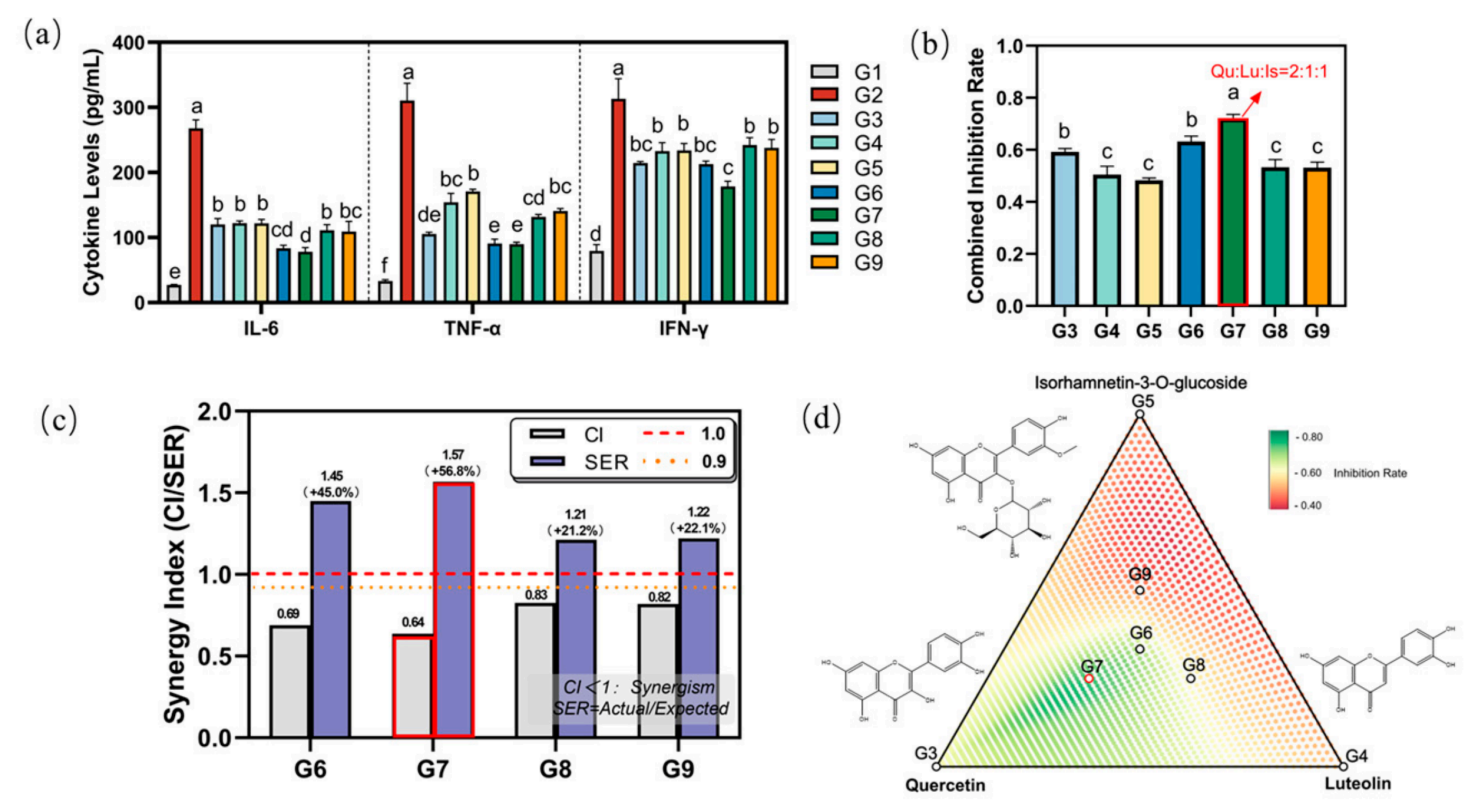
Screening and quantitative synergistic analysis of the optimal ternary formulation in vivo. (**a**) Effects of individual monomers (G3–G5) and their ternary combinations (G6–G9) on serum pro-inflammatory cytokine levels at a constant total dose of 50 mg/kg. (**b**) The combined inhibition rates across different formulations. The G7 group (Qu:Lu:Is = 2:1:1) showed the highest efficacy. (**c**) Synergistic analysis using the Combination Index (CI) and Synergy Enhancement Ratio (SER). The G7 exhibited the most synergism (CI = 0.64, SER = 1.57). (**d**) Ternary contour plot mapping component ratios to combined inhibition rates, indicating the optimal formulation space. Data are expressed as mean ± SEM (*n* = 3). Different letters indicate significant differences (*p* < 0.05).

To identify the optimal combinatorial strategy, the in vivo anti-inflammatory efficacies of individual monomers and their ternary mixtures were systematically evaluated under a constant total dose of 50 mg/kg. As shown in Figure 5a, the levels of IL-6, TNF-α, and IFN-γ were significantly suppressed in all treatment groups. Notably, the combined inhibition rate analysis revealed that the G7 formulation (Qu:Lu:Is = 2:1:1) achieved the highest overall efficacy compared to both the best-performing monomer and other combination ratios (Figure 5b).

To ascertain whether this superiority stemmed from an additive or synergistic interaction, quantitative synergistic parameters were calculated (Figure 5c). All tested ternary combinations exhibited synergistic effects (CI < 1.0). Impressively, G7 displayed the most potent synergism, characterized by the lowest CI value (0.64) and the highest SER (1.57, indicating a 57% enhancement over the theoretical additive effect). Furthermore, the ternary contour plot (Figure 5d) intuitively localized G7 within the optimal response space, corroborating the rationale of the 2:1:1 Q-dominant ratio. Consequently, G7 (designated as QLI) was selected as the optimal formulation for subsequent mechanistic investigations.

### 2.4. QLI Alleviates Fractional Radiation-Induced Physiological and Cognitive Impairments

**Figure 6:**
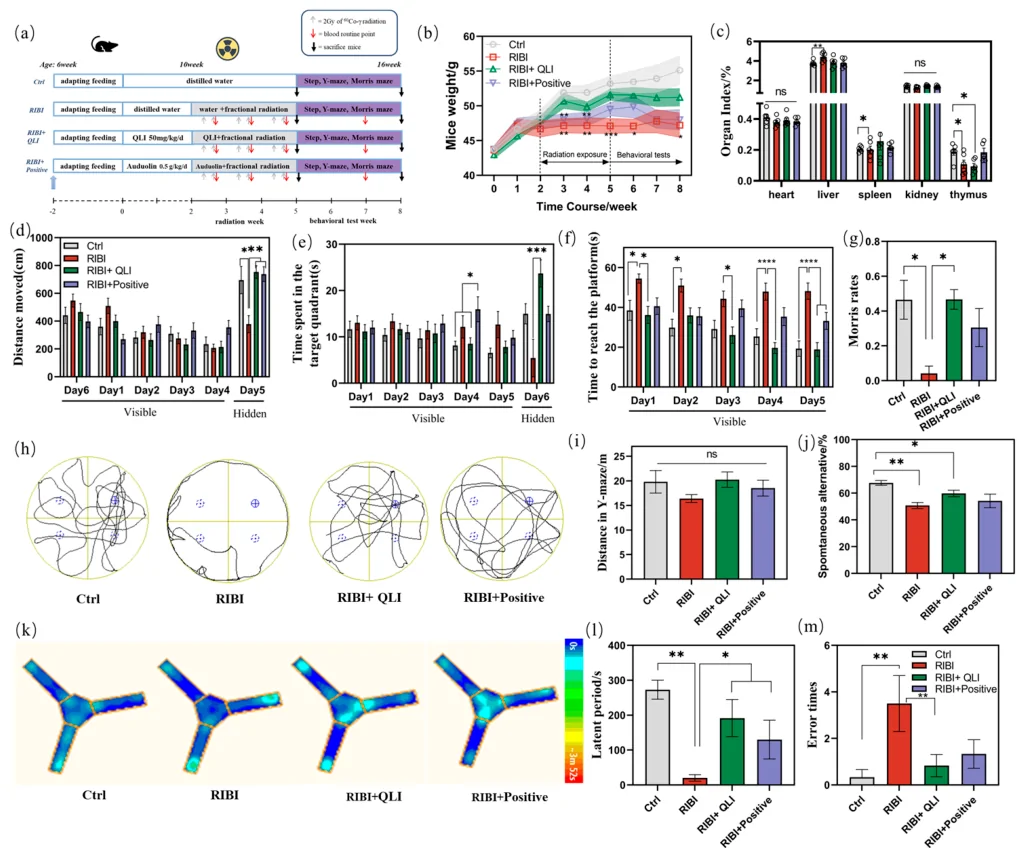
QLI alleviated fractional radiation-induced physiological and cognitive impairments in mice. (**a**) Schematic diagram of the experimental protocol; (**b**) Body weight over the 8-week period; (**c**) Organ indices of the heart, liver, kidney, thymus, and spleen; Results of the Morris water maze test, including (**d**) distance moved, (**e**) time spent in the target quadrant, (**f**) time to reach the platform and (**g**) Morris rates and (**h**) representative swimming trajectories on the probe test day; Y-maze test results, including (**i**) distance moved, (**j**) SAB and (**k**) representative movement heatmap; SDPAT results, including (**l**) the latent period and (**m**) number of errors. The data are presented as the mean ± SEM. * *p* < 0.05, ** *p* < 0.01, *** *p* < 0.001, **** *p* < 0.0001 compared between the indicated groups, ns indicates no significance.

During the first two weeks, water or drugs were chronically administered, but no significant differences were found between the groups (Figure 6b, *p* > 0.05). The weight of the Ctrl group increased steadily during the natural recovery time (weeks 5–8) with no treatment. In addition, the influence of irradiation (weeks 2–5) on body weight was difficult to determine (*p* < 0.01). Mice treated with QLI were highly resistant to cumulative radiation-induced weight loss. Unlike other reports of one-off acute radiation with a magnitude of 10 Gy causing mortality [26], the chronic cumulative radiation used in this study did not cause death, which may be related to the adaptive flexibility of the body [27]. RIBI exposure induced significant alterations in organ indices, characterized by an increased liver index and decreased spleen and thymus indices compared to the control (*p* < 0.05 or * *p* < 0.01, Figure 6c). Notably, administration of the QLI and the positive drug effectively reversed these radiation-induced pathological changes, restoring the immune and hepatic organ indices towards normal levels. The heart and kidney indices remained unaffected across all groups (ns).

The effects of radiation and drug treatment on learning and memory were assessed using the Morris water maze, Y-maze, and SDPAT. The distance travelled by the mice until the platform was not significantly different (Figure 6d, *p* > 0.05); however, in the probe test (day 6), the mice in the RIBI group swam at shorter distances and slower speeds than those in the Ctrl group (Figure 6d,f, *p* < 0.05) and the two drug groups. Compared with the RIBI+ Positive mice, the RIBI+ QLI mice exhibited shorter escape latencies (Figure 6e). The mean Morris rate was calculated (Figure 6g), and Tukey’s test revealed a significant difference between the RIBI and Ctrl/RIBI+ QLI groups (*p* < 0.05). The results of the probe test revealed differences in representative swimming paths among the groups on the 6th day of the navigation trials (Figure 6h). Compared with that of the Ctrl mice, the SAB of the RIBI mice markedly decreased (Figure 6j, *p* < 0.01), and the Ctrl and RIBI+ QLI mice performed similarly in this test. The distance in the Y-maze did not significantly differ among these groups, whereas the mean tended to decrease with increasing radiation treatment (Figure 6i,j). A warmer color temperature indicates a longer time to explore or remain (Figure 6k). In the SDPAT group, compared with the Ctrl group, the FIR group had a significantly shorter latency period and greater number of errors (*p* < 0.05). Notably, QLI treatment successfully reversed these cognitive deficits (Figure 6l,m). These results indicate that fractional radiation impairs passive avoidance efficiency, whereas QLI has strong cognition-enhancing effects comparable to those of Anduolin.

### 2.5. QLI Restores Hematopoietic Function and Neurotransmitter Homeostasis in Irradiated Mice

**Figure 7:**
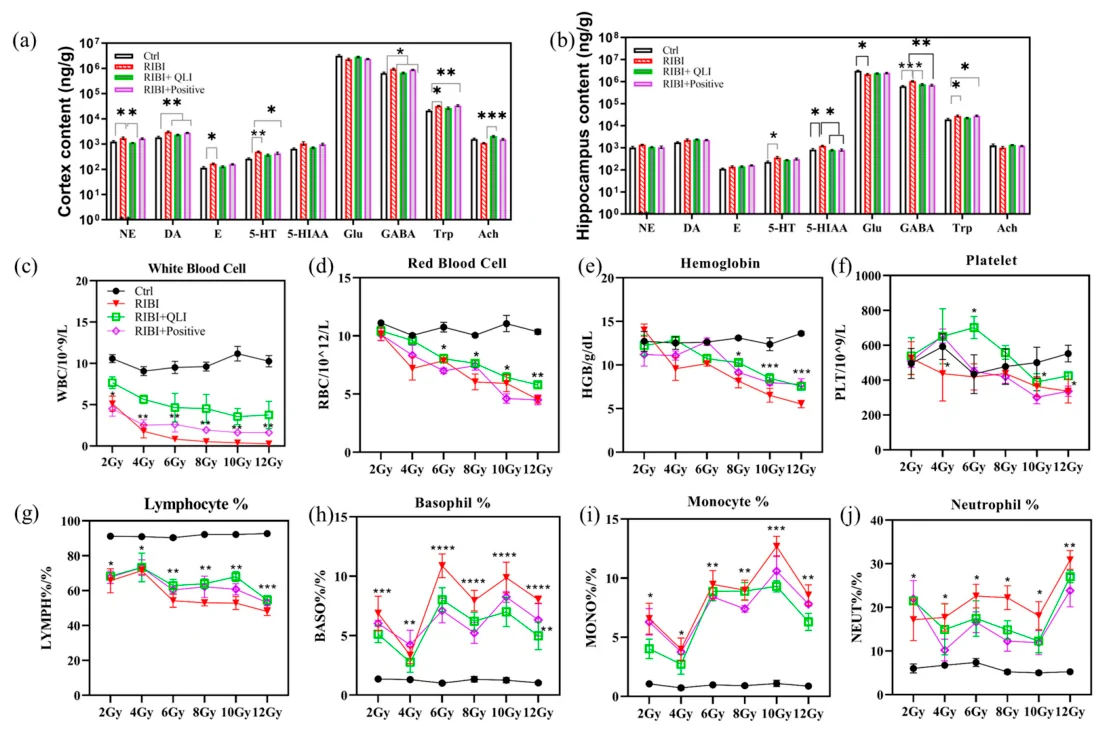
QLI restored neurotransmitter homeostasis and hematopoietic function in fractionally irradiated mice. Effect of QLI on neurotransmitter levels in the cortex (**a**) and hippocampus (**b**) of RIBI model mice; dynamic changes in routine blood indices during the cumulative fractional radiation process (from 2 Gy to 12 Gy), including white blood cell (WBC) (**c**), red blood cell (RBC) (**d**), hemoglobin (HGB) (**e**), platelet (PLT) (**f**), lymphocyte percentage (LYMPH%) (**g**), basophil percentage (BASO%) (**h**), monocyte percentage (MONO%) (**i**), and neutrophil percentage (NEUT%) (**j**). The data are presented as the mean ± SEM. * *p* < 0.05, ** *p* < 0.01, *** *p* < 0.001, **** *p* < 0.0001 compared between the indicated groups.

The levels of NE and E in the cerebral cortex were increased by fractional radiation. Compared with that in the Ctrl group, the level of DA, a precursor of NE, in the cerebral cortex was elevated by 67% following radiation (*p* < 0.01). Radiation exposure increased 5-HT levels in the cortex and hippocampus (*p* < 0.05). Compared with QLI, the positive control drug resulted in poorer inhibition of 5-HT in the cortex of the brain. 5-HIAA is the end product of the downstream reaction of Trp to 5-HT. This is the same as the cumulative trend in which the 5-HT and 5-HIAA contents increase with increasing radiation (Figure 7a). The content of Glu, an excitatory amino acid, was significantly decreased in the hippocampus (Figure 7b). Radiation exposure increased GABA levels in the cortex and hippocampus (*p* < 0.01). Trp is also activated by radiation. Compared with the positive control, QLI inhibited this activation. RIBI was involved in the loss of Ach, but among those treated with QLI or Anduolin, mice showed increased Ach production in the cortex.

Routine blood tests revealed four stages of radiation progression (Figure 7c–j). As one of the most sensitive blood cells to radiation, the WBC content is significantly reduced by FIR. After the radiation in the WBCs accumulated to 12 Gy, the body could not recover from the damage (*p* < 0.001). The WBC count in the RIBI group significantly decreased, and the degree of reduction in the RIBI+ QLI group was relatively inhibited. HGB, HCT, and PLT levels decreased as radiation exposure increased, whereas MCV, RDW, and MPV increased. QLI and positive drugs balance routine blood indices, including RBC, HGB, HCT, RDW, PLT, and MPV, to varying degrees.

### 2.6. Transcriptomic Profiling Reveals Global Gene Expression Reprogramming by QLI

**Figure 8:**
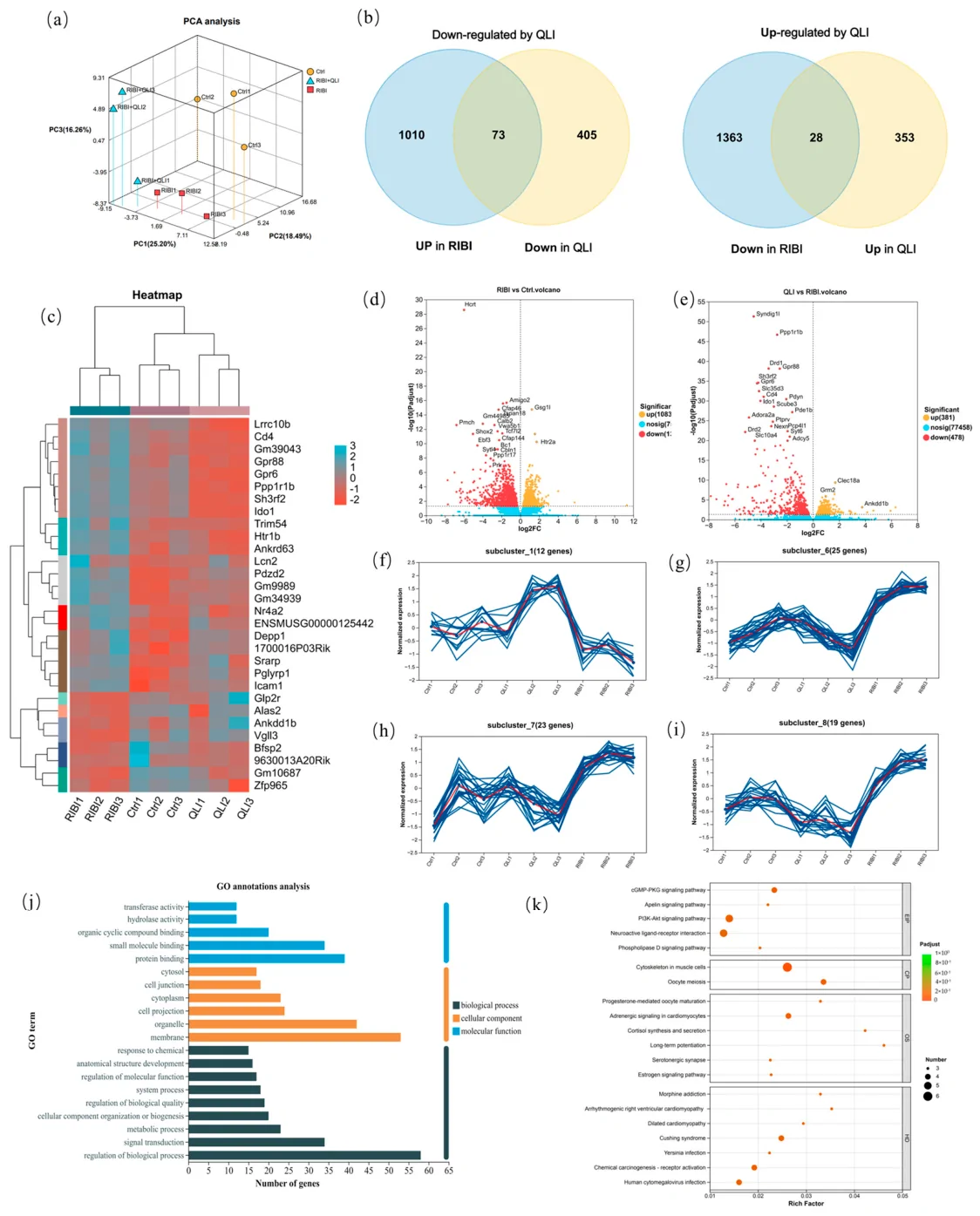
Transcriptomic profiling revealed that QLI reversed global gene expression reprogramming in fractional-irradiated mice. (**a**) 3D PCA of RNA-seq data from the Ctrl, RIBI, and RIBI+ QLI groups; (**b**) Venn diagrams illustrating the overlapping genes that are inversely regulated by QLI (left: upregulated in RIBI but downregulated by QLI; right: downregulated in RIBI but upregulated by QLI); (**c**) hierarchical clustering heatmap of the DEGs across the three experimental groups; (**d**) volcano plot displaying the differentially expressed genes (DEGs) between the RIBI and Ctrl groups; (**e**) volcano plot displaying the DEGs between the QLI and RIBI groups; (**f**–**i**) subcluster trend analysis illustrating distinct gene expression patterns (subclusters 1, 6, 7, and 8) across the Ctrl, QLI, and RIBI groups; (**j**) Gene Ontology (GO) annotation analysis of the overlapping genes, categorized into biological process, cellular component, and molecular function; (**k**) KEGG pathway enrichment analysis bubble plot, highlighting the key modulated signaling networks (such as the PI3K-Akt signaling pathway).

To elucidate the neuroprotective mechanisms of QLI at the molecular level, RNA-seq was performed on brain tissues. Principal component analysis (PCA) revealed clear spatial separation between the Ctrl, RIBI, and RIBI+ QLI groups. Notably, the transcriptomic profile of the QLI-treated group shifted away from the pathological state of the RIBI group and clustered closer to that of the normal control group (Figure 8a), indicating that QLI globally reprogrammed the aberrant gene expression induced by fractional radiation. The profound effects of radiation and subsequent therapeutic interventions are visually depicted in volcano plots (Figure 8d,e). Hierarchical clustering of differentially expressed genes (DEGs) further confirmed the distinct yet reversible expression patterns across the three experimental groups (Figure 8c).

To pinpoint the core genes rescued by the treatment, Venn diagrams were used to identify the overlapping genes that were reversely regulated by QLI. The results revealed that 28 genes whose expression was downregulated by radiation were significantly restored by QLI, whereas 73 genes whose expression was aberrantly upregulated by radiation were successfully suppressed (Figure 8b). Moreover, trend clustering analysis categorized these dynamic changes in gene expression into four specific subclusters (Figure 8f–i). The trajectories within these subclusters highlighted the rescue effect of the drug, effectively driving the pathological gene expression levels back toward physiological baselines.

Gene Ontology (GO) and Kyoto Encyclopedia of Genes and Genomes (KEGG) enrichment analyses were performed on the reversed genes to determine their biological significance. GO annotations suggested that the targeted genes were predominantly enriched in protein binding, organelle structure, membrane, and regulation of biological processes (Figure 8j). KEGG pathway analysis revealed significant enrichment in several vital neural and survival signaling cascades, including the PI3K-Akt signaling pathway, long-term potentiation (LTP), neuroactive ligand–receptor interaction, and cAMP signaling pathway (Figure 8k). These transcriptomic findings not only provide a molecular explanation for the behavioral recovery of synaptic plasticity but also highly converge with earlier network pharmacology predictions, establishing a solid multi-omics consensus on QLI’s mechanism of action against RIBI.

### 2.7. Multi-Dimensional Validation Confirms the Regulation of Key Inflammatory and Apoptotic Targets

**Figure 9:**
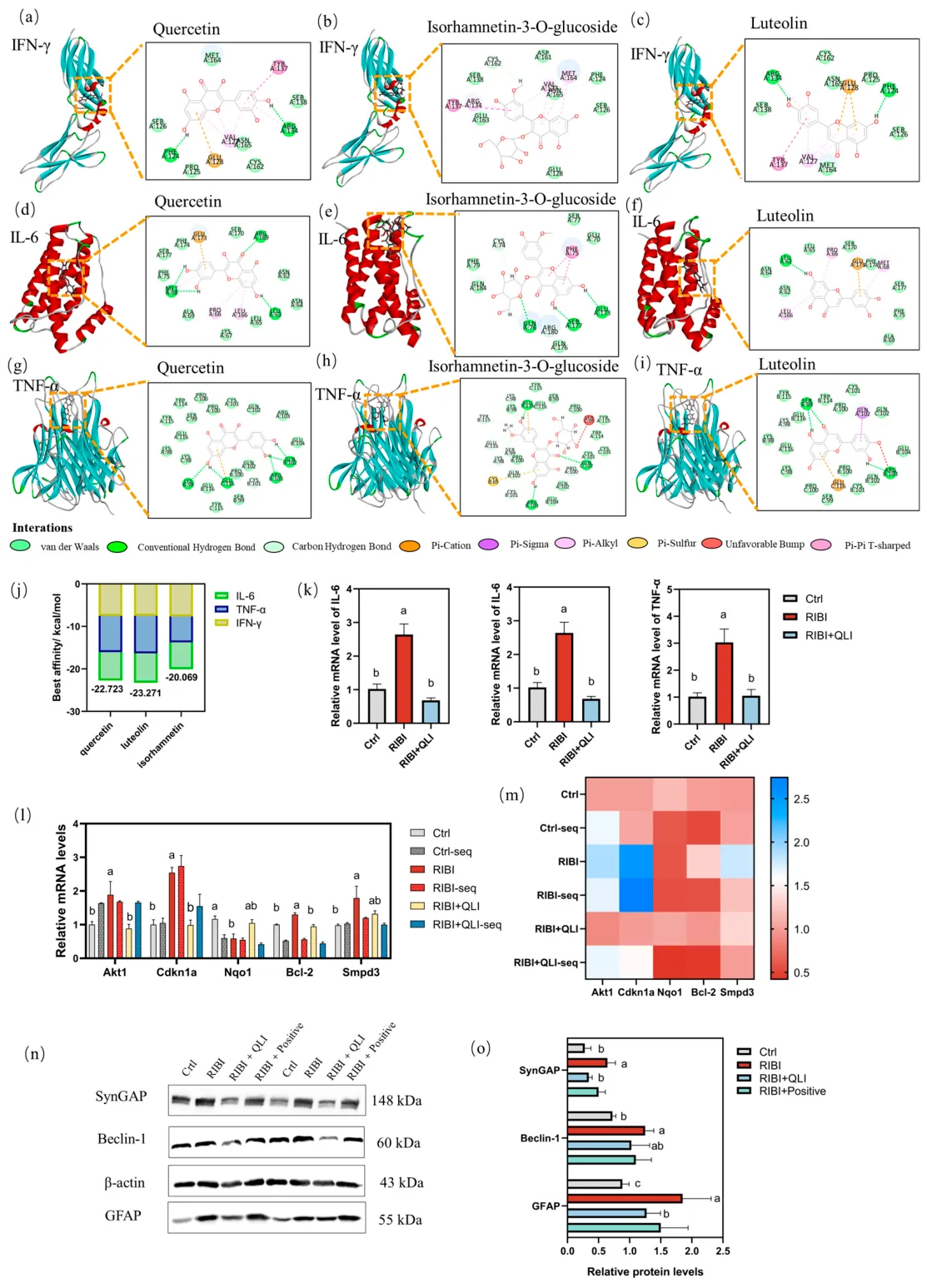
Effects of QLI on core targets associated with inflammation, apoptosis, and synaptic function. (**a**–**i**) 3D molecular docking models and corresponding 2D interaction diagrams illustrating the binding modes between active ingredients and 3 targets: IFN-γ with (**a**) quercetin, (**b**) Isorhamnetin--3-O-glucoside and (**c**) luteolin. (**d**–**f**) IL-6, (**g**–**i**) TNF-α. (**j**) Best affinity scores. 2D diagrams detail specific intermolecular interactions, such as conventional hydrogen bonds, van der Waals forces, and Pi-alkyl interactions, with key amino acid residues; (**k**) relative mRNA expression levels of the pro-inflammatory cytokines IL-6, IL-1β, and TNF-α. (**l**) Comparison of the relative mRNA expression levels of key genes (Akt1, Cdkn1a, Nqo1, Bcl-2, and Smpd3) validated by both RT-qPCR and RNA-seq; (**m**) Heatmap showing the expression patterns of the validated genes, demonstrating high consistency between the RT-qPCR and RNA-seq data; (**n**) Representative WB bands of SynGAP, Beclin-1, GFAP, and β-actin; (**o**) quantitative analysis of relative protein expression levels. The data are presented as the mean ± SEM (*n* = 3). For panels (**k**,**l**,**o**), different lowercase letters above the bars indicate statistically significant differences between groups (*p* < 0.05), whereas the same letter indicates no significant difference, as determined by one-way ANOVA followed by Tukey’s post hoc test.

Combining the results of the PPI screening and transcriptomic enrichment analysis, we performed molecular docking of three hub genes (IL-6, TNF-α, and IFNγ) with the components (quercetin, luteolin and isorhamnetin-3-O-glucoside). Nine docking models were generated from the simulations (Figure 9a–i). The 2D interaction diagrams further detail the specific binding modes, revealing intermolecular forces, including conventional hydrogen bonds, van der Waals forces, and various Pi interactions (Pi-Cation, Pi-Sigma, and Pi-Alkyl), with the key amino acid residues of the target proteins. All docked complexes exhibited strong binding affinities of <−5.0 kcal/mol, suggesting a potential structural basis for the ability of these natural compounds to directly modulate targeted inflammatory and survival proteins (Figure 9j).

To validate radiation-induced brain damage and the anti-inflammatory efficacy of QLI at the transcriptional level, we examined the expression of specific pro-inflammatory cytokines in the mouse brain using RT-qPCR. FIR significantly upregulated the mRNA levels of IL-6, IL-1β, and TNF-α (Figure 9k), indicating a severe neuroinflammatory response. Compared with the RIBI group, QLI treatment inhibited the overexpression of these inflammatory factor genes.

Furthermore, to verify the reliability of global RNA-seq data, a cohort of critical genes (Akt1, Cdkn1a, Nqo1, Bcl-2, and Smpd3) was cross-validated using RT-qPCR. As shown in Figure 9k, the distinct alphabetical statistical markers revealed highly significant transcriptional remodeling across the groups. FIR severely disrupts cellular homeostasis, leading to significant downregulation of the expression of the antioxidant gene Nqo1. Concurrently, FIR triggered a profound compensatory stress response, as evidenced by the abnormal upregulation of survival-associated genes (Akt1 and Bcl-2) and the significant activation and elevation of the anti-apoptotic protein Bcl-xl, as well as the significant increase in the expression of senescence and apoptotic markers (Cdkn1a and Smpd3). The significant differences between the RIBI and RIBI+ QLI groups confirmed that QLI effectively reversed these transcriptional disruptions, driving the hyperactive compensatory signals back toward the physiological baseline.

Western blot analysis was conducted to evaluate the core proteins associated with synaptic function, autophagy, and glial activation (Figure 9n–o). FIR markedly increased the protein expression of SynGAP (a critical negative regulator of synaptic plasticity), Beclin-1 (an indicator of autophagic/apoptotic stress), and GFAP (a marker of astrocyte hyperactivation). Consistent with the transcriptomic findings, QLI treatment significantly downregulated the pathological overexpression of these genes. Collectively, this multidimensional evidence firmly establishes that QLI exerts potent neuroprotective effects by resolving neuroinflammation, preventing apoptosis, and preserving synaptic integrity.

## 3. Discussion

FIR is a standard therapeutic modality; however, it frequently causes unavoidable collateral damage to the central nervous system, manifesting as cognitive decline and physiological dysfunction. Natural formulas have attracted attention because of their multi-target safety profiles. In this study, we suggested that the flavonoid-rich botanical extract-QLI effectively mitigates RIBI phenotypes in a mouse model. By integrating network pharmacology, RNA-seq, and multidimensional experimental validation, we propose a mechanistic model wherein QLI protects the brain by normalizing gene expression, resolving neuroinflammation, and attenuating stress-induced compensatory responses.

A critical pharmacokinetic consideration for neuroprotective agents is their ability to cross the blood–brain barrier (BBB). While native, bulky flavonoid glycosides often exhibit limited BBB penetrability, their highly lipophilic aglycones demonstrate robust BBB permeation capabilities [28]. Extensive in vivo pharmacokinetic and tissue distribution studies have repeatedly confirmed that orally or systemically administered flavonoid monomers, such as Quercetin and Luteolin, can effectively cross the BBB and accumulate in brain tissues at nanomolar to micromolar pharmacologically relevant concentrations [29,30]. These established concentrations correspond well with the threshold required to neutralize localized oxidative stress and suppress neuroinflammatory cascades in the central nervous system.

Behavioral and physiological impairments are the primary indicators of RIBI. In this study, the SAB was decreased by fractional radiation. Another study reported a similar SA reduction due to cumulative electromagnetic radiation [31]. Bekal et al. [32] reported that SA was not significantly affected by 0.5 Gy, but 1 Gy and 3 Gy irradiation significantly affected short-term memory. Mice that received 2 Gy × 6 fractionated doses rarely retained spatial memory, with low Morris rates, whereas irradiated mice that were administered QLI showed memory protection. The SDPAT revealed that, compared with the Ctrl group, the RIBI group had significantly decreased latency and increased error times. Similar findings have been reported for a single dose of acute irradiation with 15 Gy ^60^Co rays [33]. Raber et al. [34] reported that a single dose of 10 Gy had a similar effect on approximately ten 2 Gy fractions, all of which fell below the threshold known to cause evident tissue breakdown. Moreover, a modest radiation dose led to significant cognitive impairment associated with hippocampal function, which was associated with both cell proliferation and immature neuron reduction [35].

To elucidate the molecular basis of this recovery, we utilized an integrated multi-omics approach. First, UHPLC-Q-Orbitrap MS/MS analysis successfully identified 138 chemical constituents, establishing a material foundation for QLI that is predominantly characterized by flavonoids (e.g., quercetin, nobiletin, naringenin) and phenolic acids. These components are well documented for their potent antioxidant and anti-inflammatory properties. Anchored by this defined chemical profile, network pharmacology revealed 52 candidate bioactive components that interact with multiple protein targets involved in RIBI. Pathway enrichment analysis of the reversed genes closely aligned with our network predictions, indicating mainly the PI3K–Akt signaling, apoptosis, and neuroactive ligand–receptor interaction pathways. This high consistency between in silico predictions and in vivo transcriptomics suggests a plausible molecular network for the efficacy of QLI. However, we acknowledge that these bioinformatics predictions represent proposed pathways rather than definitively proven single-target mechanisms.

With blood cells serving as the most responsive indicators for assessing the effects of radiation, hematopoietic disturbances can be recognized before the onset of symptoms. In a recent study conducted by Güngördü [36], individuals who were exposed to ionizing radiation in a hospital for less than a decade exhibited decreases in WBC and neutrophil counts. Furthermore, RDW and lymphocyte counts significantly increased among individuals exposed to IR. Moreover, Wolff et al. [37] reported statistically significant decreases in WBC, RBC, HGB, HCT, MCV, PLT, and polys (*p* < 0.05) in mice for both the 4 Gy and 8 Gy doses of IR. As expected, cumulative radiation exposure resulted in significant reductions in the WBC, RBC, HGB, HCT, and PLT counts, along with increases in the MCV, RDW, and MPV. Importantly, treatment with QLI balanced these hematological indices, ameliorating the adverse effects of radiation to varying degrees. Given the crucial role played by brain TMs in regulating mood, emotional states, and behavior, there is profound interest in understanding how spaceflight affects these TMs. In a previous space experiment, Cosmos 1129 showed a reduction in NE and 5-HT levels within the hypothalamic nuclei of rats. However, DA levels remained relatively unchanged during this period [38]. Our study identified five monoamine TMs (NE, DA, E, 5-HT, and 5-HIAA), three amino TMs (Glu, GABA, and Trp) and Ach. Increased serotonin levels impair learning, whereas serotonin reuptake inhibitors improve brain function [39]. Clinically, a significant change in 5-HIAA is regarded as the most specific biomarker of carcinoid tumors, migraines, Alzheimer’s disease, and hydrocephalus [40]. Radio-induced perturbations of the central serotonergic system have been known for many years. For example, a study on rats that received neutron-gamma rays at doses of 4 Gy and 7 Gy revealed an increase in 5-HIAA levels in all regions of the brain and a decrease in the levels of DA and its metabolite in the striatum [41]. In agreement with the results of a study on space flight biosatellites [42], the DA level did not significantly change in the hippocampus. Regional differences were also observed in the present study. Compared with the hippocampus, the cortex is more sensitive to FIR stress-induced NE and E surges. Simulated spaceflight complex environments are characterized by microgravity, noise, circadian disturbances, isolation, and confinement, excluding radiation [43,44]. In these investigations, rats exhibited depression-like behaviors, accompanied by a reduction in 5-HT, DA, Glu, and Ach levels and an increase in GABA levels [45]. Trp is a precursor of 5-HT, and stress-induced release of free fatty acids from storage amplifies Trp uptake into the brain; therefore, we hypothesize that QLI intervention may reduce the level of 5-HT in the brain by lowering the level of the amino acid Trp [46].

During spaceflight or radiation therapy, the inflammatory response can lead to nuclear pyknosis in neurons and astrocytes, potentially causing neuronal apoptosis and necrosis [47]. Exposure to 4He ion irradiation at doses of 42 Gy and 168 cGy resulted in decreased levels of cortical markers for neurons and dendrites, which was associated with altered performance in the passive avoidance test [48]. Inflammatory factors, such as IL-1β, IL-6, TNF-α, NF-κB, and other cytokines, are involved in degenerative processes following radiation [33,49]. Natural polysaccharides derived from food downregulate inducible NO synthase and NF-κB levels to suppress pro-inflammatory cytokine expression [50]. Our RT-qPCR and Western blot analyses revealed a trend: FIR induced abnormal upregulation of survival-associated markers (Akt1 and Bcl-2), and specifically, the anti-apoptotic protein Bcl-xl was highly activated and significantly increased in the model group. Concurrently, markers of senescence and autophagic stress (Cdkn1a, Smpd3, and Beclin-1) were also elevated. We interpret this phenomenon as a compensatory stress response. In the face of oxidative and inflammatory stress induced by radiation, brain cells hyperactivate survival signals to counter apoptosis [51]. Interestingly, QLI treatment did not further stimulate the expression of these survival genes but rather normalized their expression levels back to the control baseline level. While our experimental data clearly demonstrated this normalization trend, we hypothesize that the active components of QLI directly neutralize upstream inflammatory and oxidative triggers. By resolving the primary stressor, QLI potentially relieves the cells from the need to maintain an overactive compensatory state, thereby restoring true cellular homeostasis while successfully downregulating actual stress markers such as Beclin-1 [52]. Furthermore, neuroinflammation and astrocyte hyperactivation are intimately linked to synaptic dysfunction in RIBI [53]. Our experimental data confirmed that FIR induced persistent activation of astrocytes, as indicated by elevated GFAP protein expression. Crucially, this neurotoxic microenvironment coincided with the abnormal accumulation of SynGAP, a critical negative regulator of synaptic plasticity [54]. Excessive upregulation of SynGAP strongly suppresses synaptic transmission and LTP formation, which mechanistically explains the severe spatial memory deficits observed in the behavioral tests [55]. Our findings demonstrate that QLI effectively normalized both GFAP and SynGAP levels. Consequently, we propose that by dampening the inflammatory cascade and restraining glial overactivation, QLI alleviates pathological suppression of synaptic plasticity and successfully rescues cognitive function.

Despite these promising findings, certain limitations exist in our current experimental design. Specifically, an independent healthy control group receiving only the QLI formulation (non-irradiated) was not included. Although the individual dietary flavonoids (quercetin, luteolin, and isorhamnetin-3-O-glucoside) possess extensively documented safety profiles and exhibit no overt baseline toxicity or adverse neuro-behavioral effects in healthy rodents at comparable doses [56,57], the absence of this control group limits our ability to completely rule out subtle baseline transcriptomic modulations induced by the exact ternary combination. Furthermore, the current study lacks comprehensive pharmacokinetic (PK) profiles and systematic toxicological assessments of the exact ternary formulation. Therefore, future studies incorporating diverse animal models, detailed PK tracking, and rigorous long-term safety evaluations are essential to fully validate the translational and clinical viability of QLI. Although we identified core therapeutic targets using transcriptomics and validated them at the tissue level, the brain is a highly heterogeneous organ. Specific cell-type responses, such as distinguishing the exact transcriptional changes in reactive astrocytes versus those in vulnerable neurons, require further investigation. Future studies utilizing single-cell RNA sequencing or conditional gene-knockout animal models will help map the precise cellular targets of QLI [58]. These findings highlight the potential of natural multi-target formulas as a viable therapeutic strategy for mitigating the neurological side effects associated with radiotherapy. Ultimately, to advance these preclinical findings toward translational applications, evaluating QLI as a concurrent adjuvant therapy in clinical radiotherapy settings could provide a scientifically grounded strategy to reduce dose-limiting neurological toxicities and improve patient quality of life.

## 4. Materials and Methods

### 4.1. Preparation of XW and Monomers

The XW formulation consists of seven medicinal materials in the following exact weight ratio: 60 parts of *Rehmanniae Radix Praeparata* (Shu Di Huang), 48 parts of *Ligustri Lucidi Fructus* (Nu Zhen Zi), 48 parts of *Cuscutae Semen* (Tu Si Zi), 24 parts of *Cervi Cornu Degelatinatum* (Lu Jiao Shuang), 72 parts of *Astragali Radix* (Huang Qi), 72 parts of *Poria* (Fu Ling), and 24 parts of *Citri Reticulatae Pericarpium* (Chen Pi). All botanical materials were purchased from Beijing Tongrentang Co., Ltd. (Beijing, China, Batch No. 223112203). The raw materials were authenticated in strict accordance with the monographs and standards specified in the *Pharmacopoeia of the People’s Republic of China*. To prepare the XW extract, the raw materials were mixed according to the specified ratio and subjected to a standardized two-step aqueous decoction process. For the first extraction, an 8-fold volume of water (solvent-to-solid ratio of 8:1, *v*/*w*) was added to the mixture, which was then boiled for 1 h. The decoction was collected, and the remaining residue was subjected to a second extraction with a 6-fold volume of water (6:1, *v*/*w*), boiling for 0.5 h. The decoctions from both extractions were combined and allowed to stand overnight to precipitate large insoluble particles. The supernatant was subsequently collected and filtered through three layers of gauze. The filtered liquid was then concentrated under vacuum at a pressure of −0.06 to −0.08 MPa. Finally, the concentrated extract was dried under reduced pressure (−0.06 to −0.08 Pa) at a carefully controlled temperature of ≤80 °C to yield the final dry extract powder. The calculated extraction yield of the final lyophilized XW powder was approximately 23.5% (*w*/*w*) relative to the crude starting materials. Voucher specimens (Voucher No. HIT-XW-202308) have been deposited at the laboratory herbarium of Harbin Institute of Technology (Harbin, China) for future reference. Quercetin (Q4951), luteolin (L9283), and isorhamnetin-3-O-glucoside (ST9H9BC16FFA) (purity > 90%) were purchased from Sigma-Aldrich (St. Louis, MO, USA). The QLI formulation was prepared by physically mixing the three monomers at a mass ratio of 2:1:1. For in vivo administration, QLI was suspended in 0.5% (*w*/*v*) sodium carboxymethyl cellulose (CMC-Na) to ensure stable suspension.

### 4.2. Network Pharmacology

#### 4.2.1. Screening of Bioactive Components in XW

The Traditional Chinese Medicine Systems Pharmacology platform (TCMSP) (https://ngdc.cncb.ac.cn/databasecommons/database/id/4096, accessed on 25 August 2026) was used for systemic pharmacological studies [22]. We searched for the chemical constituents of seven herbs (SDH, NZZ, TSZ, HQ, FL, CP, and LJS), and preliminary screening of the active ingredients was carried out on the basis of two ADME attribute values: oral bioavailability (OB) ≥30% and drug-likeness (DL) ≥0.18 for screening of the pharmacokinetically favorable candidate compounds and their protein targets. Target names were converted to uniform standard UniProt IDs using the UniProt protein database (http://www.uniprot.org).

#### 4.2.2. RIBI-Related Targets

In the GeneCards (https://www.genecards.org) and DisGeNet (https://www.disgenet.org/) databases, we used the keywords “radiation damage,” “brain damage,” and “radiation-induced brain injury” to retrieve RIBI-related targets. Clinical drug targets for radiation diseases were searched in the DrugBank database (https://www.drugbank.ca).

#### 4.2.3. XW Gene‒Disease Target Gene PPI Network

Using the Venn diagram package, we integrated the drug- and RIBI-related targets. Intersecting targets were submitted to the STRING11.0 database (https://string-db.org) to model the protein-protein interaction (PPI) network using the Molecular Complex Detection (MCODE) plugin (Cytoscape 3.7.2).

#### 4.2.4. Active Ingredients of XW Disease Target Gene Pathways

CytoScape 3.7.2 built-in tools were used to analyze the network topology parameters of the active ingredients and targets, including degree, betweenness, and closeness. The core targets and active ingredients that exert pharmacological effects should be determined on the basis of these parameters.

### 4.3. UHPLC-Q-Orbitrap MS/MS Analysis of XW

The chemical composition of the XW extract was systematically characterized using a UHPLC system coupled to a Q Exactive Plus hybrid quadrupole-Orbitrap mass spectrometer (Thermo Fisher Scientific, Waltham, MA, USA). Briefly, 20 mg of XW powder was ultrasonically extracted in 1 mL of 70% methanol for 30 min at room temperature, centrifuged at 10,000 rpm for 5 min, and filtered through a 0.22 μm nylon membrane prior to analysis. Chromatographic separation was achieved on a Kinetex C18 column (2.1 × 100 mm, 1.7 μm) maintained at 40 °C, with an injection volume of 10 μL. The mobile phase consisted of ultrapure water (Watsons, Guangzhou, China) containing 0.1% formic acid (Thermo Fisher, Phase A) and acetonitrile (Thermo Fisher, Phase B) containing 0.1% formic acid, delivered via gradient elution at a flow rate of 0.3 mL/min. The complete gradient program was set as follows: 0–11 min, 5–95% B; 11–12.5 min, 95% B; 12.5–12.6 min, 95–5% B; 12.6–15 min, 5% B, yielding a total run time of 15 min. Mass spectrometric data were acquired using a heated electrospray ionization (HESI) source in both positive and negative ion modes under a Full MS/data-dependent MS2 (ddMS2) scanning strategy to tentatively identify the bioactive constituents.

The HESI source parameters were configured as follows: sheath gas flow rate, 9 arb; spray voltage, 3.0 kV; capillary temperature, 320 °C; auxiliary gas heater temperature, 30 °C; and S-lens RF level, 55. For the Full MS acquisition, the mass range was set to *m*/*z* 100–1200 with a resolving power of 70,000, an automatic gain control (AGC) target of 1e6, and a maximum injection time of 100 ms. For the ddMS2 scans, the resolving power was 35,000, with an apex trigger of 3–9 s, a dynamic exclusion time of 8 s, and stepped normalized collision energies of 20, 40, and 60.

Compound annotation was performed by matching the exact mass, isotopic patterns, and MS/MS fragmentation spectra against standard mass spectral databases (mzCloud and mzVault). The limited availability of reference spectra—all database-based identifications in this study are considered tentative unless further supported by authentic reference standards or complementary analytical evidence.

### 4.4. Animals and Grouping

Six-week-old male SPF Kunming mice (40 ± 2 g) were purchased from the Experimental Animal Center of Vital River (Beijing, China). The temperature was maintained at 23 °C with 12-h light and 12-h dark cycles. A preliminary screening experiment was conducted to determine the optimal anti-inflammatory formulation. A total of 27 male Kunming mice were randomly assigned to nine groups (*n* = 3 per group). Groups G1 and G2 served as the blank control and model control, respectively. Groups G3–G5 were treated with individual monomers (Quercetin, Luteolin and Isorhamnetin-3-O-glucoside, 50 mg/kg/d). Groups G6–G9 were treated with different ternary combinations of the three monomers and standardized at 50 mg/kg. Specifically, the groups were defined as follows: G6 (1:1:1), G7 (2:1:1), G8 (1:2:1), and G9 (1:1:2).

The optimal ternary formulation (G7, 2:1:1) was selected and designated as QLI. To further evaluate its neuroprotective efficacy and underlying mechanisms, a second batch of mice (*n* = 48) was established and randomly divided into four groups. Ctrl (control), RIBI (model), RIBI+ QLI (QLI treatment) and RIBI+ Positive (Anduolin treatment). All animal experiments were approved by the Peking University Laboratory Animal Center (Beijing, China). The approval number was IACUC-2021-006.

### 4.5. Radiation Treatment and the Use of Drugs

The mice were fixed in a resin cage with stomata and transported to the Beijing Peking University Irradiation Center in an animal transport box for ^60^Co-γ-ray whole-body irradiation. The Ctrl mice were also transported but were not irradiated. The irradiation regimen was 2 Gy (at a dosage rate of 1 Gy/min) of ^60^Co-γ twice a week for three consecutive weeks, and the final cumulative dose was 12 Gy. The mice in the RIBI+ QLI group received intragastric QLI (50 mg/kg/d). Those in the RIBI+ positive group were administered Anduolin capsules (0.5 g·kg^−1^; Fujian Zhongan Pharmaceutical Co. Ltd., Z10970016, Fujian, China) by gavage once a day [59,60], which is a well-established proprietary Chinese medicine clinically approved for mitigating radiation-induced toxicities, and were selected as the positive control to provide a robust pharmacological benchmark [61,62]. The standard clinical human equivalent dose is 3.84 g/day, according to the manufacturer’s clinical package insert. Based on established allometric dose scaling guidelines utilizing body surface area normalization [63], this translates to an equivalent murine dose of 0.5 g·kg^−1^. Although this 0.5 g·kg^−1^ dose is numerically higher than the 50 mg/kg dose of QLI, it represents the gross weight of the capsule, which includes crude plant matrices and essential excipients. In contrast, the QLI formulation consists of highly enriched active monomers with purity approaching 100%, making their respective biologically active doses pharmacologically comparable. An equal volume of distilled water was administered to the control and RIBI groups. Behavioral tests were conducted from 9:00 to 12:00, five weeks after drug administration.

### 4.6. Quantification of Serum Inflammatory Cytokines by ELISA

To evaluate the in vivo anti-inflammatory efficacies of the individual monomers and their ternary combinations during the preliminary screening phase, the serum levels of key pro-inflammatory cytokines were quantified. At the end of the experimental period, blood samples were collected from the mice and centrifuged at 3000 rpm for 15 min at 4 °C to separate the serum. The concentrations of Interleukin-6 (IL-6), Tumor Necrosis Factor-alpha (TNF-α), and Interferon-gamma (IFN-γ) in the serum were measured using specific commercial Enzyme-Linked Immunosorbent Assay (ELISA) kits, including the QuantiCyto^®^ Mouse IL-6 ELISA kit, QuantiCyto^®^ Mouse TNF-α ELISA kit, and QuantiCyto^®^ Mouse IFN-γ ELISA kit (all purchased from Neobioscience Technology Co., Ltd., Shenzhen, China). All assay procedures were conducted in strict accordance with the manufacturer’s instructions. The optical density (OD) absorbance of each well was measured at 450 nm using a microplate reader, and the exact cytokine concentrations were calculated based on the standard curves generated concurrently.

### 4.7. Behavioral Testing

#### 4.7.1. Morris Water Maze

After adaptive feeding, the mice were weighed and recorded weekly. The apparatus used was a swimming pool based on that described by Morris and adapted for mice [64]. The method used in this study was slightly modified from that described in our previous report [45]. The mice received five days of training (four trials per day) to acclimate to the pool and find the visible platform and then received hidden platform training (one trial) to assess spatial learning and memory. During the probe test, spatial memory retention was specifically evaluated using the Morris rate. This ratio is quantitatively defined as the number of times the mice crossed the exact location of the original target platform divided by the total number of crossings over the equivalent platform locations across all four quadrants.

#### 4.7.2. Y Maze

The symmetrical Y-maze consisted of three open arms (A, B, and C) at 120° angles to each other. The mice were placed in the center, facing a wall as the starting arm (A), and the total number (a) and number of times they entered different arms consecutively (b) were recorded within 8 min. These data were used to calculate spontaneous alternation behavior (SAB), which comprises the tendency of mice to alternate their nonreinforced choice of Y-maze arms on successive opportunities [65]. The alternations are then divided by the number of alternation opportunities, namely, the total number of arm entries minus one (Equation (1)).

SAB = b/(a − 1) × 100%,(1)

#### 4.7.3. Mouse Step-Down Passive Avoidance Test (SDPAT)

The SDPAT was used to measure memory retention and nonspatial learning and memory, as previously reported [33]. During the training session, each mouse was first placed in the box to acclimatize for 3 min, after which the copper grid was electrified (3 mA, 65 V) for 5 min. Memory retention tests were performed 24 h after the training session. Each mouse was placed on a platform with electrified bars for 5 min. The latency and error times were then calculated.

### 4.8. Routine Determination

Six mice from each group were randomly selected for routine blood testing. Each mouse was anaesthetized with 1% pentobarbital sodium, and whole blood (0.5 mL) was collected in a 1.5 mL centrifuge tube with EDTA K2 microtubes. The samples were analyzed using an automated hematology analyzer (MEK-6400, Nihon-Kohden, Tokyo, Japan).

### 4.9. Tissue Processing

After the last irradiation, the mice were deeply anaesthetized with pentobarbital sodium and decapitated to collect their brain tissues on ice. The brains were dissected into hippocampi and cortices and stored at −80 °C until analysis.

### 4.10. Neurotransmitters Measurement

Neurotransmitter (TM) levels in the hippocampus and cortex were measured using liquid chromatography‒tandem mass spectrometry (LC-MS/MS). This method was developed by Wang [66] for the simultaneous determination of DA, tryptophan (Trp), 5-hydroxytryptamine (5-HT), 5-hydroxyindoleacetic acid (5-HIAA), norepinephrine (NE), epinephrine (E), L-glutamic acid (Glu), γ-aminobutyric acid (GABA), and acetylcholine (Ach) in a single chromatographic run.

### 4.11. Transcriptomic Analysis

Total RNA was extracted from the brain tissue, and the mRNA was enriched using oligo(dT) magnetic beads. Following mRNA fragmentation, double-stranded cDNA was synthesized, end-repaired, A-tailed, and ligated to the sequencing adapters. The constructed libraries were subsequently sequenced using the Illumina HiSeq platform. The raw data were filtered to obtain high-quality clean reads, which were subsequently aligned to the reference genome. Gene expression levels were quantified, and differential expression analysis was performed using the DESeq2 R package (v 4.5.3) [67]. Differentially expressed genes (DEGs) were identified on the basis of the thresholds of a |log2-fold fold change| ≥1 and an adjusted *p* value < 0.05.

### 4.12. Molecular Docking

Molecular docking can be used to explore the interactions between macromolecules and small-molecule ligands. It is generally believed that van der Waals forces, hydrogen bonds, hydrophobic interactions, and electrostatic interactions are the main factors influencing docking studies. Molecular docking studies were conducted using DS, and the crystal structures of three targets, IL6, TNFα, and IFNγ, were obtained from the RCSB Protein Data Bank (https://www.rcsb.org). The three targets were docked with the ingredients (quercetin, isorhamnetin-3-O-glucoside, and luteolin) using the Systems Dock website (http://systemsdock.com).

### 4.13. Quantitative RT-PCR Assay

Total RNA was extracted from mouse brains using TRIzol reagent (Ambion, Texas, USA). cDNA was synthesized using the QuantiTect^®^ Rev. Transcription Kit (Qiagen, Duesseldorf, Germany). Real-time PCR was performed via a realplex2 real-time PCR system (Eppendorf, Hamburg, Germany). The primers used in this study are listed in Table A1.

### 4.14. Western Blot

The proteins were separated by SDS-PAGE and transferred onto PVDF membranes. The membranes were blocked with 5% nonfat milk and incubated overnight at 4 °C with primary antibodies against GFAP (ab7260; Abcam (Cambridge, MA, USA); 1:10,000), SynGAP (ab77235; Abcam; 1:5000), Beclin-1 (#3495; Cell Signaling (Danvers, MA, USA); 1:1000), and β-actin (GB15003; Servicebio; 1:3000). Following incubation with horseradish peroxidase-conjugated secondary antibodies (Servicebio, Wuhan, China), the membranes were developed using enhanced chemiluminescence. The BCA protein assay kit (PC0020), stripping buffer (SW3020) and fast blocking (SW3012) were purchased from Beijing Solarbio Science & Technology Co., Ltd., Beijing, China. The band intensities were quantified using ImageJ (version 1.54d, National Institutes of Health, Bethesda, MD, USA) to determine the protein intensities.

### 4.15. Statistical Analysis

All the results are expressed as the mean ± standard error of the mean (SEM). Other data were evaluated using one-way analysis of variance followed by Tukey’s post hoc tests (IBM SPSS Statistics 25.0) for multiple comparisons, with GraphPad Prism 8.0.2 for drawing. Differences were considered statistically significant at *p* < 0.05 and *p* < 0.01. For specific bar charts, different lowercase letters above the bars indicate statistically significant differences between groups (*p* < 0.05), whereas the same letter indicates no significant difference.

## 5. Conclusions

This study provides preclinical experimental evidence suggesting that the flavonoid-rich botanical formulation, QLI, has the potential to protect against fractional radiation-induced brain injury. In our current model, QLI administration was associated with the amelioration of cognitive deficits, improved peripheral hematopoiesis, and more stable neurotransmitter balance. Mechanistically, our findings indicate that the neuroprotective effects of QLI are linked to broad transcriptomic modulations. These transcriptomic associations potentially involve resolving neuroinflammation, relieving the pathological suppression of synaptic plasticity, and normalizing the radiation-induced compensatory overshoot of survival and apoptotic signaling pathways. These findings highlight the potential of natural multi-target formulas as a promising experimental approach for mitigating the neurological side effects of radiotherapy. Despite these promising findings, the clinical relevance of QLI must be interpreted with caution; further studies incorporating diverse animal models, detailed pharmacokinetic tracking, and systematic toxicological evaluations are essential to establish definitive causal mechanisms and validate the translational viability of QLI.

## Figures and Tables

**Table 1:** Basic information of key targets of XW against the RIBI.

Rank	UniProt ID	Gene Symbol	Protein Name	Degree
1	P31749	AKT1	RAC-αserine/threonine-protein kinase	113
2	P01375	TNF	Tumor necrosis factor	105
3	P04637	TP53	Cellular tumor antigen p53	105
4	P05231	IL6	Interleukin-6	104
5	P42574	CASP3	Caspase-3	100
6	P01584	IL1B	Interleukin-1 beta	98
7	P03372	ESR1	Estrogen receptor	97
8	P05412	JUN	Transcription factor Jun	97
9	P10415	BCL2	Apoptosis regulator Bcl-2	97
10	P35354	PTGS2	Prostaglandin G/H synthase 2	96

**Table 2:** Main active ingredients and KEGG pathways.

Class	Number	Annotation	Node Parameter		
			Degree	Betweenness Centrality	Closeness Centrality
Drug Component	C	Quercetin	154	0.324	0.582
	D2	Kaempferol	57	0.033	0.431
	NZZ6	Syringaresinol iglucoside_qt	39	0.075	0.455
	NZZ1	Taxifolin	29	0.033	0.431
	B	beta-Sitosterol	28	0.015	0.389
	D1	Isorhamnetin	24	0.008	0.391
	HQ9	Formononetin	16	0.024	0.397
	CP4	Nobiletin	16	0.017	0.395
	CP1	Naringenin	13	0.026	0.393
	HQ5	7-O-Methylisomucronulatol	12	0.012	0.382
	SDH1	Stigmasterol	9	0.011	0.370
	E	Hederagenin	9	0.003	0.360
KEGG Pathway	hsa05200	Pathways in cancer	45	0.080	0.468
	hsa04151	PI3K-Akt signalling pathway	30	0.039	0.407
	hsa05418	Fluid shear stress and atherosclerosis	27	0.030	0.391
	hsa04933	AGE-RAGE signalling pathway in diabetic complications	27	0.021	0.382
	hsa05166	Human T-cell leukemia virus 1 infection	26	0.025	0.380
	hsa04210	Apoptosis	21	0.017	0.377
	hsa04066	HIF-1 signalling pathway	19	0.015	0.372
	hsa05202	Transcriptional mis-regulation in cancer	17	0.011	0.355
	hsa05160	Hepatitis C	16	0.012	0.357
	hsa04064	NF-kappa B signalling pathway	14	0.010	0.386

**Table 3:** Identification of the main chemical constituents in XW.

No.	Compound	Formula	Adduct	*m*/*z*	PPM	Main Class
1	2-Phenylglycine	C_8_H_9_NO_2_	[M+H]^+^	152.07	−2.2	Amino acids and their derivatives
2	Val-Leu	C_11_H_22_N_2_O_3_	[M+H]^+^	231.16	−2.63	Amino acids and their derivatives
3	Osmanthuside H	C_19_H_28_O_11_	[M-H]^−^	431.16	−1.92	Phenolic acids
4	Daidzein-8-C-glucoside	C_21_H_20_O_9_	[M-H]_−_	415.10	−1.85	Isoflavones
5	Chrysin6-C-glucoside8-C-arabinoside	C_26_H_28_O_13_	[M+H]^+^	549.16	2.03	Flavonoid carbon glycosides
6	Isoacteoside	C_29_H_36_O_15_	[M-H]^−^	623.20	−2.38	Phenolic acids
7	Isorhamnetin-3-O-glucoside	C_22_H_22_O_12_	[M-H]^−^	477.10	−0.83	Flavonols
8	Calycosin	C_16_H_12_O_5_	[M-H]^−^	283.06	−7.47	Isoflavones
9	Quercetin	C_15_H_10_O_7_	[M-H]^−^	301.03	6.56	Flavonols
10	Comanthoside B	C_23_H_22_O_12_	[M-H]^−^	489.10	0.11	Flavones
11	Datiscetin	C_15_H_10_O_6_	[M-H]^−^	285.04	−1.69	Flavonols
12	Nobiletin	C_21_H_22_O_8_	[M+H]^+^	403.14	−0.31	Flavones
13	Glycycoumarin	C_21_H_20_O_6_	[M-H]^−^	367.12	−2.19	Coumarins
14	5,7-Dihydroxy-3,6-dimethoxyflavone	C_17_H_14_O_6_	[M+H]^+^	315.09	−7.06	Flavonols
15	Tangeretin	C_20_H_20_O_7_	[M+H]^+^	373.13	−1.28	Flavones
16	3-oxoglycyrrhetinic acid	C_30_H_44_O_4_	[M+H]^+^	469.33	−3.63	Triterpenes
17	13-HOTrE	C_18_H_30_O_3_	[M-H]^−^	293.21	−1.46	Free fatty acids
18	Betulinic acid	C_30_H_48_O_3_	[M-H]^−^	455.35	−0.95	Triterpenes
19	Auraptene	C_19_H_22_O_3_	[M-H]^−^	297.15	−1.39	Coumarins
20	Phthalic anhydride	C_8_H_4_O_3_	[M+H]^+^	149.02	−2.68	Carboxylic acid anhydrides

Abbreviation: *m*/*z*, Mass-to-charge ratio; PPM, Parts per million.

## Data Availability

The raw data supporting the conclusions of this article will be made available by the authors on request.

## Abbreviations

The following abbreviations are used in this manuscript:

5-HIAA	5-hydroxyindoleacetic acid
5-HT	5-hydroxytryptamine
Ach	Acetyl-choline
ANOVA	Analysis of variance
BP	Biological process
CC	Cellular component
CP	Citrus Reticulata
Ctrl	Control
DA	Dopamine
DEG	Differentially expressed gene
DL	Drug-likeness
E	Epinephrine
FIR	Fractional ionizing radiation
FL	Poria Cocos
FRT	Fractionated radiotherapy
GABA	γ-aminobutyric acid
GFAP	Glial fibrillary acidic protein
Glu	L-glutamic acid
GO	Gene Ontology
HCT	Hematocrit
HGB	Hemoglobin
HQ	Astragalus membranaceus
IL-1β	Interleukin-1 beta
IL-6	Interleukin-6
IFN-γ	Interferon gamma
IR	Ionizing radiation
KEGG	Kyoto Encyclopedia of Genes and Genomes
UHPLC-Q-Orbitrap MS/MS	Ultra-High Performance Liquid Chromatography-Quadrupole-Orbitrap Tandem Mass Spectrometry
LJS	Cornu Cervi Degelatinatum
LTP	Long-term potentiation
MCV	Mean corpuscular volume
MF	Molecular function
MPV	Mean platelet volume
NE	Norepinephrine
NZZ	Fructus Ligustri Lucidi
OB	Oral bioavailability
PCA	Principal component analysis
PLT	Platelet
PPI	Protein–protein interaction
QLI	A ternary mixture of Quercetin, Luteolin, and Isorhamnetin-3-O-glucoside
RBC	Red Blood Cell
RDW	Red cell distribution width
RIBI	Radiation-induced brain injury
RNA-seq	RNA-sequencing
RT-qPCR	Reverse transcription quantitative polymerase chain reaction
SAB	Spontaneous alternation behavior
SDH	Rehmanniae Radix Praeparata
SDPAT	Step-down passive avoidance test
SEM	Standard error of the mean
SPF	Specific pathogen-free
TCM	Traditional Chinese Medicine
TCMSP	Traditional Chinese Medicine Systems Pharmacology platform
TMs	Neurotransmitters
TNF-α	Tumor necrosis factor-alpha
Trp	Tryptophan
TSZ	Cuscutae Semen
WBC	White Blood Cell

## Appendix A

### Appendix A.1

**Table A1:** Primer sequence of mRNA.

Name		Primer Sequence (5′-3′)	Tm	Product Size (bp)
Akt	F	AGATGGACTCAAGAGGCAGGAAG	62.3	85
Akt	R	TACTCAAACTCGTTCATGGTCACAC	61.7	
Il6	F	GAGAGGAGACTTCACAGAGGATACC	60.1	131
Il6	R	TCATTTCCACGATTTCCCAGAGAAC	60.8	
Bcl2	F	TACGAGTGGGATGCTGGAGATG	62.9	119
Bcl2	R	TCAGGCTGGAAGGAGAAGATGC	60.6	
Il-1β	F	GCAACTGTTCCTGAACTCAACT	60.7	89
Il-1β	R	ATCTTTTGGGGTCCGTCAACT	61.4	
Tnf-α	F	CCCTCACACTCAGATCATCTTCT	62.0	140
Tnf-α	R	GCTACGACGTGGGCTACAG	60.3	
Smpd3	F	ACACGACCCCCTTTCCTAATA	60	129
Smpd3	R	GGCGCTTCTCATAGGTGGTG	62.6	
Nrf2	F	ATAGAGCAGGACATGGAGCAAG	58.8	207
Nrf2	R	AAGGAAATGTGGACCACAGTTG	59.0	
Cdkn1a	F	GAGTAGGACTTTGGGGTCTCCT	60.6	134
Cdkn1a	R	CACAGGTCTGAGCAATGTCAAG	59.5	
Nqo1	F	TGCCTACACATATGCTGCCAT	59	206
Nqo1	R	CAATGCTGTAAACCAGTTGAGGT	58.9	
β-actin-f	F	CTGAGAGGGAAATCGTGCGT	60.0	208
β-actin-r	R	CCACAGGATTCCATACCCAAGA	61.3	

### Appendix A.2

**Table A2:** In silico screened candidate components of XW.

Herb	Serial Number	Molecule Name	Molecule Structure	OB	DL
Rehmanniae Radix Praeparata	A	Sitosterol		36.91	0.75
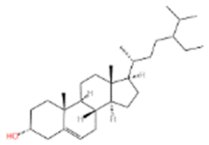
Rehmanniae Radix Praeparata	SDH1	Stigmasterol		43.83	0.76
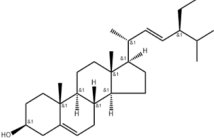
Fructus Ligustri Lucidi	B	β-sitosterol		36.91	0.75
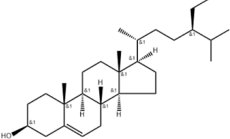
Fructus Ligustri Lucidi	D2	Kaempferol		41.88	0.24
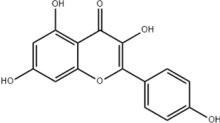
Fructus Ligustri Lucidi	NZZ1	Taxifolin		57.84	0.27
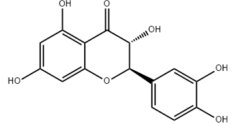
Fructus Ligustri Lucidi	NZZ2	Lucidumoside D		48.87	0.71
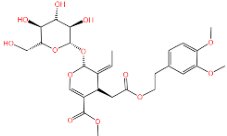
Fructus Ligustri Lucidi	NZZ3	Lucidumoside D_qt		54.41	0.47
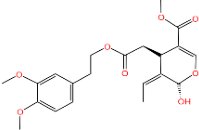
Fructus Ligustri Lucidi	NZZ4	(20S)-24-ene-3β,20-diol-3-acetate		40.23	0.82
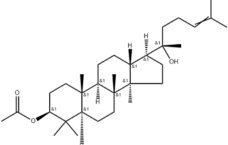
Fructus Ligustri Lucidi	NZZ5	Eriodictyol		71.79	0.24
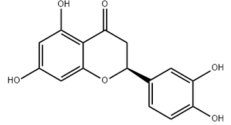
Fructus Ligustri Lucidi	NZZ6	Syringaresinol diglucoside_qt		83.12	0.8
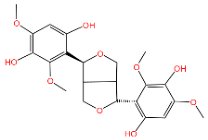
Fructus Ligustri Lucidi	NZZ7	Lucidusculine		30.11	0.75
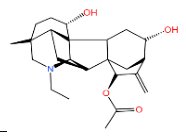
Fructus Ligustri Lucidi	NZZ8	Olitoriside		65.45	0.23
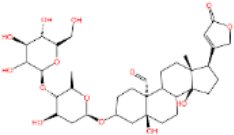
Fructus Ligustri Lucidi	NZZ9	Olitoriside_qt		103.23	0.78
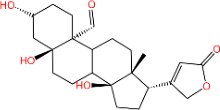
Fructus Ligustri Lucidi	NZZ10	Luteolin		36.16	0.25
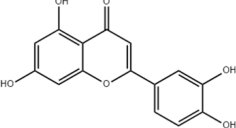
Fructus Ligustri Lucidi	C	Quercetin		46.43	0.28
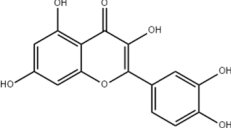
Fructus Ligustri Lucidi	TSZ1	Sesamin		56.55	0.83
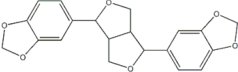
Fructus Ligustri Lucidi	TSZ2	NSC63551		39.25	0.76
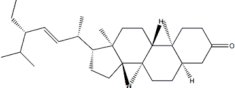
Fructus Ligustri Lucidi	D1	Isorhamnetin		49.6	0.31
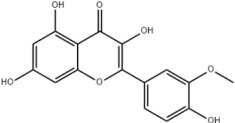
Fructus Ligustri Lucidi	B	beta-sitosterol		36.91	0.75
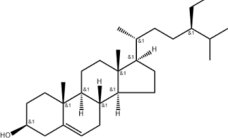
Fructus Ligustri Lucidi	D2	Kaempferol		41.88	0.24
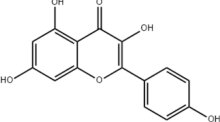
Fructus Ligustri Lucidi	TSZ3	Campest-5-en-3beta-ol		37.58	0.71
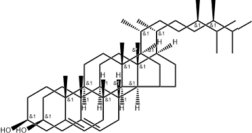
Fructus Ligustri Lucidi	TSZ4	Isofucosterol		43.78	0.76
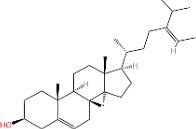
Fructus Ligustri Lucidi	TSZ5	Matrine		63.77	0.25
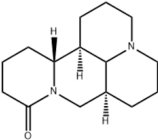
Fructus Ligustri Lucidi	TSZ6	Sophoranol		55.42	0.28
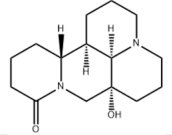
Fructus Ligustri Lucidi	TSZ7	Cholesterol		37.87	0.68
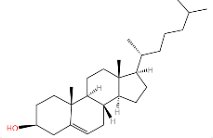
Fructus Ligustri Lucidi	C	Quercetin		46.43	0.28
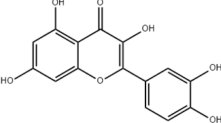
Hedysarum Multijugum Maxim.	HQ1	Mairin		55.38	0.78
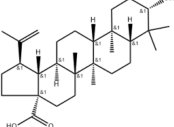
Hedysarum Multijugum Maxim.	HQ2	Jaranol		50.83	0.29
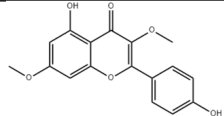
Hedysarum Multijugum Maxim.	E	Hederagenin		36.91	0.75
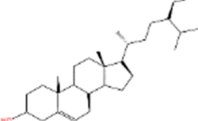
Hedysarum Multijugum Maxim.	HQ3	(3S,8S,9S,10R,13R,14S,17R)-10,13-Dimethyl-17-[(2R,5S)-5-propan-2-yloctan-2-yl]^−^2,3,4,7,8,9,11,12,14,15,16,17-dodecahydro-1H-cyclopentaphenanthren-3-ol		36.23	0.78
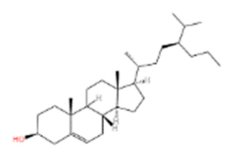
Hedysarum Multijugum Maxim.	D1	Isorhamnetin		49.6	0.31
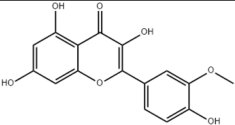
Hedysarum Multijugum Maxim.	HQ4	3,9-di-O-Methylnissolin		53.74	0.48
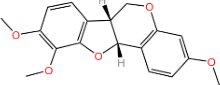
Hedysarum Multijugum Maxim.	HQ5	7-O-Methylisomucronulatol		36.74	0.92
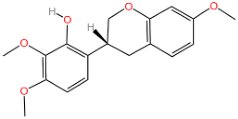
Hedysarum Multijugum Maxim.	HQ6	9,10-Dimethoxypterocarpan-3-O-β-D-glucoside		64.26	0.42
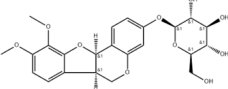
Hedysarum Multijugum Maxim.	HQ7	Methylnissolin		64.26	0.42
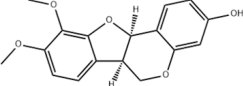
Hedysarum Multijugum Maxim.	HQ8	Bifendate		31.1	0.67
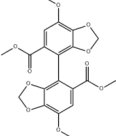
Hedysarum Multijugum Maxim.	HQ9	Formononetin		69.67	0.21
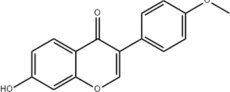
Hedysarum Multijugum Maxim.	HQ10	Calycosin		47.75	0.24
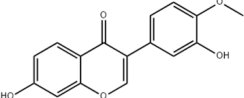
Hedysarum Multijugum Maxim.	D2	Kaempferol		41.88	0.24
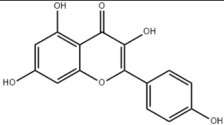
Hedysarum Multijugum Maxim.	HQ11	Folic acid		68.96	0.71
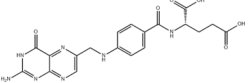
Hedysarum Multijugum Maxim.	HQ12	1,7-Dihydroxy-3,9-dimethoxy pterocarpene		39.05	0.48
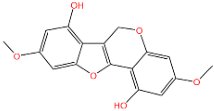
Hedysarum Multijugum Maxim.	C	Quercetin		46.43	0.28
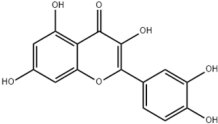
Poria Cocos(Schw.) Wolf.	FL1	16α-Hydroxydehydrotrametenolic acid		30.93	0.81
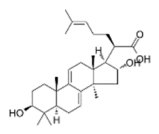
Poria Cocos(Schw.) Wolf.	FL2	Trametenolic acid		38.71	0.8
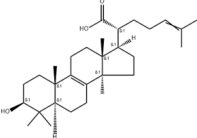
Poria Cocos(Schw.) Wolf.	FL3	Cerevisterol		37.96	0.77
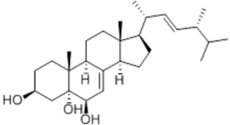
Poria Cocos(Schw.) Wolf.	FL4	Ergosta-7,22E-dien-3beta-ol		43.51	0.72
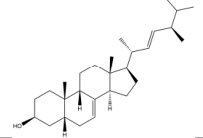
Poria Cocos(Schw.) Wolf.	FL5	Ergosterol peroxide		40.36	0.81
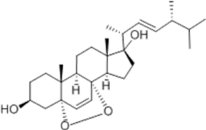
Poria Cocos(Schw.) Wolf.	E	Hederagenin		36.91	0.75
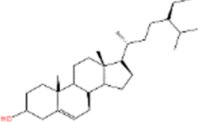
Citrus Reticulata	A	Sitosterol		36.91	0.75
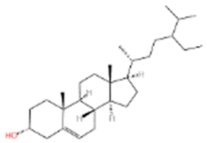
Citrus Reticulata	CP1	Naringenin		59.29	0.21
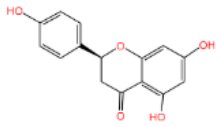
Citrus Reticulata	CP2	5,7-dihydroxy-2-(3-hydroxy-4-methoxyphenyl) chroman-4-one		47.74	0.27
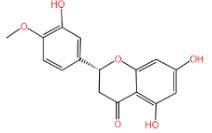
Citrus Reticulata	CP3	Citromitin		86.9	0.51
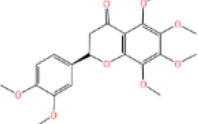
Citrus Reticulata	CP4	Nobiletin		61.67	0.52
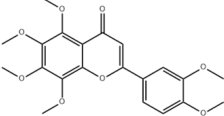
Cornu Cervi Degelatinatum	LJS1	Aspartic acid		79.74	0.02
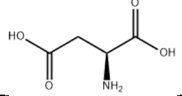
Cornu Cervi Degelatinatum	LJS2	Glycine		48.74	0
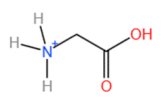
Cornu Cervi Degelatinatum	LJS3	D-Alanine		85.17	0.01
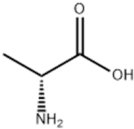
Cornu Cervi Degelatinatum	LJS4	D-Serin		98.47	0.01
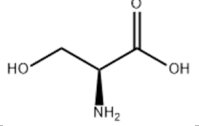
Cornu Cervi Degelatinatum	LJS5	L-Isoleucine		59.05	0.02
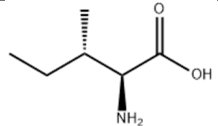
Cornu Cervi Degelatinatum	LJS6	L-Threonine		73.52	0.01
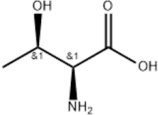
Cornu Cervi Degelatinatum	LJS7	Uracil		42.53	0.02
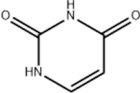
Cornu Cervi Degelatinatum	LJS8	Phenylalanine		41.62	0.04
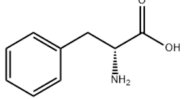
Cornu Cervi Degelatinatum	LJS9	Histidine		53.18	0.03
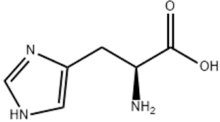
Cornu Cervi Degelatinatum	LJS10	Proline		77.57	0.01
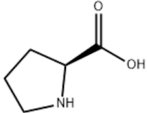
Cornu Cervi Degelatinatum	LJS11	Arginine		47.64	0.03
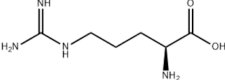
Cornu Cervi Degelatinatum	LJS12	Valine		53.33	0.01
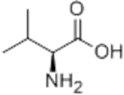

The compounds listed were selected based on pharmacokinetic prediction thresholds of oral bioavailability (OB) ≥30% and drug-likeness (DL) ≥0.18. These parameters suggest theoretical pharmacokinetic feasibility and druggability, but do not definitively demonstrate biological activity in vivo. Additionally, amino acid constituents derived from the animal-based material Cornu Cervi Degelatinatum (LJS1–LJS12) were retained independent of the DL threshold due to their endogenous physiological relevance and distinct active absorption pathways.
